# Unveiling Scale-Dependent Statistical Physics: Connecting Finite-Size and Non-Equilibrium Systems for New Insights

**DOI:** 10.3390/e28010099

**Published:** 2026-01-14

**Authors:** Agustín Pérez-Madrid, Iván Santamaría-Holek

**Affiliations:** 1 Departament de Física de la Matèria Condensada, Facultat de Física, Universitat de Barcelona, Martí i Franquès 1, 08028 Barcelona, Spain; 2 Unidad Multidisciplinar de Docencia e Investigación-Facultad de Ciencias, Universidad Nacional Autónoma de México Campus Juriquilla, Querétaro 76230, Mexico; isholek.fc@gmail.com

**Keywords:** scale-dependent effective temperature, non-equilibrium systems, quantum confinement

## Abstract

A scale-dependent effective temperature emerges as a unifying principle in the statistical physics of apparently different phenomena, namely quantum confinement in finite-size systems and non-equilibrium effects in thermodynamic systems. This concept effectively maps these inherently complex systems onto equilibrium states, thereby enabling the direct application of standard statistical physics methods. By offering a framework to analyze these systems as effectively at equilibrium, our approach provides powerful new tools that significantly expand the scope of the field. Just as the constant speed of light in Einstein’s theory of special relativity necessitates a relative understanding of space and time, our fixed ratio of energy to temperature suggests a fundamental rescaling of both quantities that allows us to recognize shared patterns across diverse materials and situations.

## 1. Introduction

Non-equilibrium systems deviate from thermal equilibrium, causing their macroscopic properties (such as temperature, pressure, and volume) to vary over time, often due to external forces like shear, electric fields, or chemical gradients. In such systems, these macroscopic properties may not be globally well-defined, so effective or local quantities are often used instead. Systems far from equilibrium, such as those under strong driving forces, as well as glassy or active matter systems, are a particular class where the standard fluctuation–dissipation theorem (FDT) no longer applies [1,2]. In these systems exhibiting behaviors far distinct from equilibrium, the concept of effective temperature is often introduced to modify or extend the FDT and provide a generalized framework for characterization [3,4,5,6,7,8].

While traditionally studied through different lenses, the breakdown of bulk thermodynamics in finite-size systems presents conceptual challenges akin to those in non-equilibrium systems, where standard macroscopic definitions are inadequate. When combined with quantum confinement effects, finite-size systems exhibit unique thermodynamic and quantum behaviors, as both finite-size corrections and energy quantization contribute to deviations from bulk properties. Crucially, their thermodynamic properties lose extensivity, meaning that they are no longer proportional to the system size [9,10,11].

Both far-from-equilibrium and finite-size systems exhibit unique traits, including amplified fluctuations, non-equilibrium correlations, and deviations from classical thermodynamic assumptions, which challenge traditional methods of analysis and necessitate innovative approaches to understand their complex and often system-specific behaviors. Effective temperature offers a generalized, well-explored framework for characterizing non-equilibrium and non-classical systems, providing crucial insights [12,13,14,15].

In this review, we propose a method based on the concept of a scale-dependent effective temperature, which can be applied to both far-from-equilibrium systems and finite-size systems. This approach highlights the shared underlying physics of these systems [12,13,14,15].

The generation mechanism for this effective temperature in both non-equilibrium and certain quantum-confined systems is detailed throughout the remainder of the paper, commencing after Section 2. We have ensured a robust presentation by including dedicated sections that rigorously test our theoretical framework against confirmatory experimental evidence for every application. We emphasize that, to execute the mentioned tests, we have detailed in each case the derivation of the characteristic properties usually measured experimentally. This introduces some necessary technical content; however, it can be omitted, allowing the reader to proceed directly to the results.

## 2. Thermal Scaling

The thermal scaling that will be discussed in this section is associated to the existence of a characteristic length scale that arises either from non-equilibrium conditions imposed by the presence of external forces driving the system under question or due to extreme confinement. We will first introduce the non-equilibrium case as it is the most intuitive form to realize and understand the origin of effective temperatures and, as a consequence, of the thermal scaling.

### 2.1. External Forces and Non-Equilibrium Systems

Given an external force F=−∇V, we can associate a local length scale with this force, defined by the following relation

(1) $$l∼\frac{\Delta V}{\left|\nabla V\right|},$$

such that the magnitude of the gradient of the potential, |∇V| can be expressed as

(2) $$\left|\nabla V\right|∼\frac{\Delta V}{l}.$$

Here, ΔV refers to the global variation of V(x) between the system’s extremes. On the other hand, according to the mean value theorem of the differential calculus, for a well-behaved function V(x), it is possible to express ΔV=αL, where *L* represents the length of the system, and α is the value of |∇V| in some point of the system. Based on this, we have

(3) $$\left|\nabla V\right|∼\alpha \frac{L}{l}.$$

Analyzing the ratio $\frac{L}{l}$ allows us to determine the dynamical regime of the system. When L≪l, the external force acts as a linear perturbation. Conversely, when the condition l≪L is met, the system shifts into a far-from-equilibrium nonlinear regime, which is particularly intriguing. In this context, we define an effective temperature, θ, as a modified version of the control temperature, *T*, incorporating an additive correction:

(4) θ=T+ΔT,

where the correction term ΔT must be functionally dependent on the ratio $\frac{L}{l}$, expressed as ΔT=f(L/l). This effective temperature represents the state in which the system behaves as if it were in thermal equilibrium with a hypothetical reservoir at θ. Likewise, paralleling the structure of Equation (10), the variable θ is related to a scaling of *E* and *T*:

(5) $$\frac{E}{T}=\frac{\hat{E}}{\theta }=\frac{\hat{E}}{T+\Delta T},$$

where

(6) $$\hat{E}=E\left(1+\frac{\Delta T}{T}\right).$$

The presence of the external force, as clearly shown in Equation (6), introduces an additional, generally non-thermal, agitation energy. This phenomenon has a direct implication for the Boltzmann probability distribution, which will be discussed in the following subsection.

Moreover, a dimensional analysis indicates that this correction term must also rely on an effective mass, $m^{*}$, and a relaxation time, τ, associated with energy dissipation, such that

(7) $$f\left(\frac{L}{l}\right)∼\frac{1}{k_{B}}\frac{1}{m^{*}}{\left(\tau \alpha \frac{L}{l}\right)}^{2}=\frac{1}{k_{B}}\frac{1}{m^{*}}{\left(\tau |\nabla V|\right)}^{2},$$

where use has been made of Equation (3). This leads us to infer that the temperature θ serves as a characteristic feature of far-from-equilibrium systems, regardless of their specific nature.

The key physical basis for this scaling is that the required multiplicative scaling factor is no longer constant, but depends on the absolute temperature:

(8) $$\hat{E}=E\cdot \lambda \left(T\right)\;\mathrm{where}\;\lambda \left(T\right)=1+\frac{\Delta T}{T}.$$

This implies that the statistical equivalence of the two systems (*E* and $\hat{E}$) is maintained, but only via a temperature-dependent energy renormalization. This form is often an *effective (phenomenological) description* used to analyze systems dominated by an *intrinsic thermal offset*, such as a non-zero critical temperature in phase transitions. A parallelism can be seen if we set $\Delta T=-T_{c}$. Hence, the effective temperature becomes $\theta =T-T_{c}$, which plays the role of the *reduced temperature*, measuring the temperature deviation from the critical temperature $T_{c}$. However, along the present work we will explore applications focused on non-equilibrium and confined systems rather than critical phenomena.

### 2.2. Effective Temperatures and Boltzmann Statistic

In statistical mechanics, the probability of a system being in a certain state is given by the Boltzmann distribution,

(9) $$P∼\exp\left(-E/k_{B}T\right),$$

where *E* is the energy of this state, $k_{B}$ is the Boltzmann constant, and *T* is the temperature. Rescaling both energy and temperature can lead to equivalent descriptions that retain the same statistical properties. By introducing a dimensionless scale variable such as ε=E/kT, we can analyze the fundamental statistical relationships within systems independent of their specific energy and temperature scales. This type of rescaling, when applied appropriately within the Boltzmann factor, can then be adapted to construct a size-dependent thermo-statistical description by incorporating how the characteristic energy and temperature scales themselves vary with system size.

Such that the invariant speed of light (a length–time ratio) in Einstein’s relativity necessitates the relative scaling of space and time, a relationship quantified by the Lorentz transformations. Similarly, our guiding principle or a demand for a consistent theoretical framework, relying on the invariance of the Boltzmann factor points to a fundamental rescaling of energy and temperature, which helps us uncover universal behaviors shared by diverse materials and structures. Hence, the inherent invariance of statistical mechanics under the simultaneous scaling of both energy and temperature is revealed. Thus, the following scaling relation involving the temperature θ can be established:

(10) $$\frac{E}{k_{B}T}=\frac{\hat{E}}{k_{B}\theta },$$

where $\hat{E}$ is the rescaled energy. It is worth noting that an alternative form of Equation (10):

(11) $$E=\frac{T}{\theta }\hat{E},$$

has a counterpart in the literature on thermally activated processes [16]. This suggests that the topics explored here generate significant interest.

Furthermore, we have established that introducing an effective temperature θ is mathematically equivalent to incorporating a chemical potential μ, leading to the energy transformation $\hat{E}=E-m^{*}\mu$. This equivalence becomes apparent when rewriting Equation (10) as

(12) $$\frac{E}{k_{B}T}=\frac{\hat{E}}{k_{B}\theta }=\frac{\hat{E}+m^{*}\mu }{k_{B}T}.$$

Here, μ takes on an analogous role to θ. Later on, we will discuss an example in which Equation (12) comes into play explicitly.

The previous discussions suggests that we can write the Boltzmann probability in the following form

(13) $$\hat{P}∼\exp\left(-\hat{E}/k_{B}\theta \right),$$

or equivalently

(14) $$\hat{P}∼\exp\left[-\left(E-m^{*}\mu \right)/k_{B}\theta \right].$$

Here, $m^{*}\mu$ is the excess energy that must be supplied or removed to account for the difference between the system’s true energy (*E*) and the rescaled energy ($\hat{E}$). These equations constitute our central result. In particular, Equation (13) recovers Boltzmann statistics with an effective thermal energy that implicitly incorporates non-equilibrium and confinement effects.

It is convenient to clarify that thermal properties, like the internal energy *u* or the specific heat $c_{p}$, are usually determined using the continuous distribution of energy characterized by a density of states ρ(E). In the general form, we have

(15) u(T)=∫u(T,E)ρ(E)dE,

where u(T,E) is the spectral energy. The same quantity should be obtained if the calculation is performed using the rescaled energy $\hat{E}$, whose conjugate temperature is θ

(16) $$\hat{u}\left(\theta \right)=\int \hat{u}\left(\theta ,\hat{E}\right)\hat{\rho }\left(\hat{E}\right)d\hat{E},$$

where now $\hat{\rho }\left(\hat{E}\right)$ is the density of states that depends on the rescaled energy and $\hat{u}\left(\theta ,\hat{E}\right)$ the modified spectral energy. While $u\left(T\right)=\hat{u}\left[\theta \left(T\right)\right]$, the density of states ρ(E) and $\hat{\rho }\left(\hat{E}\right)$ and the spectral quantities u(T,E) and $\hat{u}\left(\theta ,\hat{E}\right)$ are, in general, not equal. However, the total number of contributing energy states has to be the same in both cases. This point will be illustrated and discussed more thoroughly when analyzing confined radiation phenomena in Section 6 and thereafter.

## 3. Brownian Motion in the Presence of External Forces

In this section, we detail the method for determining the effective temperature θ in systems subject to forces derived from external potentials. In addition, we show how this effective temperature enables the formulation of a generalized fluctuation–dissipation theorem (FDT). Furthermore, we establish that the adiabatic elimination of fast variables can be carried out as if the system were undergoing linear fluctuations, despite the dynamics not being strictly linear.

### 3.1. Effective Temperature and Fokker–Planck Dynamics [17,18]

For systems governed by Fokker–Planck dynamics, an explicit expression for this effective temperature can be derived by following the steps outlined below.

To illustrate, let us examine the Fokker–Planck equation corresponding to the normalized distribution function, f(x,u,t), of velocities, *u*, and positions, *x*, of the particles of a one-dimensional Brownian gas in a heat bath at a temperature of $T_{0}$:

(17) $$\begin{array}{cc} \frac{\partial }{\partial t}f\left(\Gamma ,t\right) & =-u\nabla f+\nabla \left\{\frac{V(x,t)}{m}\right\}\frac{\partial }{\partial u}f+\\ \; & \xi \left(\frac{k_{B}T_{0}}{m}\frac{\partial^{2}}{\partial u^{2}}f+\frac{\partial }{\partial u}uf\right), \end{array}$$

where V(x,t) represents an external potential that may vary with position and time, and ξ is a dissipative coefficient. Furthermore, for more concise notation, we have define Γ=(x,u) and ∇=∂/∂x. According to Equation (17), the dynamics of the variables *x* and *u* are generally coupled, meaning that the adiabatic elimination of the fast variable *u* is not permissible in most cases. Nevertheless, it is reasonable to express

(18) $$f\left(\Gamma ,t\right)=\phi_{x}\left(u,t\right)\rho \left(x,t\right),$$

where $\phi_{x}\left(u,t\right)$ is the conditional probability density and ρ(x,t)=m∫uf(Γ,t)du is the configurational probability density. This factorization shows that the velocity *u* behaves as a slave variable to *x*, which remains effectively frozen on timescales exceeding its characteristic relaxation time $\xi^{-1}$ (i.e., $\xi^{-1}$ is the relaxation time of the fast, inertial degrees of freedom). In this regime, an effective temperature field can be defined as

(19) $$\theta \left(x,t\right)\equiv \frac{m}{k_{B}}\int u^{2}\phi_{x}\left(u,t\right)du.$$

For timescales longer than $\xi^{-1}$, these fast modes have relaxed, and their influence can be effectively represented by an effective temperature—namely, the temperature of the effective thermal bath acting on the slow configurational degrees of freedom.

From Equation (17), we derive a hierarchy of coupled evolution equations for the conditional raw moments of the fast degrees of freedom [17], which can be truncated at the second moment for times greater than the characteristic time $\xi^{-1}$. Thus, as it is illustrated in Ref. [18], we obtain the expression of the effective temperature:

(20) $$k_{B}\theta \left(x,t\right)=k_{B}T+\frac{1}{m}{\left(\frac{\nabla V(x,t)}{\xi }\right)}^{2}.$$

The second term on the right-hand side includes the gradient of the external potential in a form that resembles that predicted by dimensional analysis, Equation (7), provided we make the identification $\tau ∼\xi^{-1}$. In addition to this, we also obtain a closed expression of the first raw moment which is the current

(21) $$J\left(x,t\right)=-D\left(x,t\right)\left\{\nabla \rho \left(x,t\right)+\frac{\rho (x,t)}{k_{B}\theta \left(x,t\right)}\nabla \Phi \left(x,t\right)\right\},$$

where now $D\left(x,t\right)=\left(k_{B}\theta \left(x,t\right)/m\right)\xi^{-1}$ is the bare effective diffusion coefficient and $\Phi \left(x,t\right)=V\left(x,t\right)+k_{B}\theta \left(x,t\right)$ is an effective potential. Note that the effective temperature alters the potential, which is no longer V(x,t), but instead, a new expression that includes θ(x,t) as an additive term. This effective temperature introduces an entropic contribution to the current which becomes manifest by rewriting Equation (21) as

(22) $$\begin{array}{c} J\left(x,t\right)=-D\left(x,t\right)\left\{\nabla \rho (x,t)+\right.\\ \left.\frac{\rho (x,t)}{k_{B}\theta \left(x,t\right)}\nabla V\left(x,t\right)+\rho \left(x,t\right)\ln\theta \left(x,t\right)\right\}. \end{array}$$

Since the explicit dependencies of $\phi_{x}\left(u,t\right)$ are not known a priori, it is convenient to adopt a mean-field approach as a working framework. To this end, we average over the inhomogeneities of the energy landscape and thus, define the marginal distribution of velocities, $\phi \left(u,t\right)\equiv \int \phi_{x}\left(u,t\right)\rho \left(x,t\right)dx$, satisfying the modified Rayleigh equation

(23) $$\begin{array}{cc} \frac{\partial \phi }{\partial t} & =\\ \; & \xi \frac{\partial }{\partial u}\left\{\left(u+\frac{\langle \nabla V\rangle }{\xi }\right)\phi +\frac{k_{B}T}{m}\frac{\partial \phi }{\partial u}\right\}, \end{array}$$

in which 〈∇V〉=∫ρ(x,t)∇V(x,t)dx is the average force. Note that Equation (23) is valid to zero order in the expansion

(24) $$\phi_{x}\left(u,t\right)=\phi \left(u,t\right)+\frac{\left|\nabla V\right|}{L\xi^{2}}\delta \phi \left(u,t\right)+o\left(\nabla V^{2}\right).$$

To facilitate the solution of Equation (23), we rewrite it in a more convenient convective form as

(25) $$\begin{array}{cc} \frac{\partial \phi }{\partial t} & -\left(\xi u+\langle \nabla V\rangle \right)\frac{\partial \phi }{\partial u}=\\ & \frac{k_{B}T\xi }{m}\frac{\partial^{2}\phi }{\partial u^{2}}+\xi \phi . \end{array}$$

Here, the term $\left[-\left(\xi u+\langle \nabla V\rangle \right)\right]$ represents the drift component in the convective derivative, defined as

(26) $$\frac{d}{dt}\phi \equiv \frac{\partial }{\partial t}\phi -\left(\xi u+\langle \nabla V\rangle \right)\frac{\partial }{\partial u}\phi .$$

The drift term determines the characteristic lines, which are the paths that individual points (or probability masses) follow in *u* space over time. Therefore, an observer moving along with the drift would perceive changes in *u* according to

(27) $$u=u_{0}e^{-\xi t}-\int_{0}^{t}e^{-\xi (t-s)}\langle \nabla V\left(s\right)\rangle ds.$$

Equivalently, we can write

(28) $$u_{0}=ue^{\xi t}+\int_{0}^{t}e^{\xi s}\langle \nabla V\left(s\right)\rangle ds,$$

which is the first constant of motion. Hence, as explained in Ref. [19], using Equation (28), after some mathematical manipulations, one finds that the Gaussian

(29) $$\begin{array}{cc} \phi (u,t)= & \frac{1}{\sqrt{\frac{2\pi k_{B}T}{m}\left(1-e^{-2\xi t}\right)}}\times \\ \; & \exp\left\{-\frac{{\left[u-\left(u_{0}e^{-\xi t}-\int_{0}^{t}e^{-\xi (t-s)}\langle \nabla V\left(s\right)\rangle ds\right)\right]}^{2}}{\frac{2k_{B}T}{m}\left(1-e^{-2\xi t}\right)}\right\} \end{array}$$

is a solution of Equation (23). For times much longer than $\xi^{-1}$ and under the assumption of smooth variations in 〈∇V(t)〉, Equation (29) simplifies to

(30) $$\phi \left(u,t\right)=\frac{1}{\sqrt{\frac{2\pi k_{B}T}{m}}}\exp\left\{-\frac{{\left[u-\langle \nabla V\left(t\right)\rangle /\xi \right]}^{2}}{\frac{2k_{B}T}{m}}\right\}.$$

This equation describes a Gaussian distribution centered at u=〈∇V(t)〉/ξ with a variance of $k_{B}T/m$. This result indicates that, for sufficiently long times, the velocity distribution remains Gaussian. It shifts according to 〈∇V(t)〉 and maintains a temperature-dependent width. The second raw moment of Equation (30) is then given by

(31) $$\bar{u^{2}}=\frac{k_{B}T}{m}+{\left(\frac{\langle \nabla V(t)\rangle }{\xi }\right)}^{2}\equiv \frac{k_{B}\theta_{mf}\left(t\right)}{m},$$

this defines the mean field temperature which is the spatial average of the non-equilibrium temperature Equation (20). Therefore, we can write the generalized Smoluchowski equation

(32) $$\frac{\partial }{\partial t}\rho \left(x,t\right)=D_{mf}\nabla^{2}\rho \left(x,t\right)+\xi^{-1}\nabla [\rho \left(x,t\right)\nabla V\left(x,t\right),$$

where

(33) $$D_{mf}=\left(k_{B}\theta_{mf}/m\right)\xi^{-1}$$

is the mean-field diffusion coefficient. Neglecting the time derivative leads to the quasi-equilibrium solution of Equation (32), which is defined by the mean-field thermal energy $k_{B}\theta_{mf}$:

(34) $$\hat{\rho }\left(x\right)∼\exp\left[-\frac{V(x,t)}{k_{B}\theta_{mf}}\right].$$

This equation shows that the system recovers the Boltzmann statistics discussed in Section 2, as can be seen in Equation (13).

### 3.2. Langevin Dynamics [20]

Let us consider now how the effective temperature emerges from a stochastic point of view by analyzing the non-equilibrium case using a generalized Langevin equation.

For this case, we consider a Brownian degree of freedom *v* representing, for example, the velocity of a particle in a thermal bath whose dynamics is given by the following generalized Langevin equation

(35) $$\frac{dv}{dt}=-\int_{0}^{t}\kappa \left(t-t^{\prime }\right)v\left(t^{\prime }\right)dt^{\prime }+X\left(t\right)+F\left(t\right),$$

where $\kappa (t-t^{\prime })$ is the memory kernel related to the dissipative forces arising in the interaction between the heat bath and the Brownian degree of freedom *v*. The thermal random force F(t) is assumed Gaussian, thus having zero average 〈F(t)〉=0, and second moment [21]

(36) $$\langle F\left(t\right)F\left(t^{\prime }\right)\rangle =k_{B}T\kappa \left(t-t^{\prime }\right),$$

where now the brackets indicate an average over the realizations of the noise. In addition, as usual, we assume that

(37) 〈v(0)F(t)〉=0.

Here, we leave open the possibility that X(t) represents either a deterministic force or a noise of non-thermal character, such as an Ornstein–Uhlenbeck noise.

The usual method to solve Equation (35) is the Laplace transform, yielding

(38) $$v\left(t\right)=v\left(0\right)R\left(t\right)+\int_{0}^{t}R\left(t-t^{\prime }\right)\left[X\left(t^{\prime }\right)+F\left(t^{\prime }\right)\right]dt^{\prime },$$

where we have introduced the memory function $R\left(t\right)=L^{-1}\left\{\frac{1}{s+\hat{\kappa }\left(s\right)}\right\}$, with $\hat{\kappa }\left(s\right)$ being the Laplace transform of κ(t). To simplify the procedure, it is convenient to introduce the purely random variable $\tilde{v}\left(t\right)$:

(39) $$\tilde{v}\left(t\right)\equiv v\left(t\right)-\int_{0}^{t}R\left(t-\tau \right)X\left(\tau \right)d\tau ,$$

which is defined as the result of subtracting the motion induced by X(t), so that consequently

(40) $$\tilde{v}\left(t\right)=\tilde{v}\left(0\right)R\left(t\right)+\int_{0}^{t}R\left(t-t^{\prime }\right)F\left(t^{\prime }\right)dt^{\prime },$$

and $\tilde{v}\left(0\right)=v\left(0\right)$. By analogy with Equations (38) and (35), we infer that

(41) $$\frac{d}{dt}\tilde{v}\left(t\right)=-\int_{0}^{t}\kappa \left(t-\tau \right)\tilde{v}\left(\tau \right)d\tau +F\left(t\right),$$

which describes a stationary Gaussian process [22].

To define an effective temperature we must compute the second raw moment $\langle v{\left(t\right)}^{2}\rangle$. From (39), we find that

(42) $$\begin{array}{c} \langle v{\left(t\right)}^{2}\rangle =\langle {\tilde{v}}^{2}\left(t\right)\rangle +\\ \int_{0}^{t}\int_{0}^{t}R\left(t-\tau \right)R\left(t-\tau^{\prime }\right)\langle X\left(\tau \right)X\left(\tau^{\prime }\right)\rangle d\tau d\tau^{\prime }. \end{array}$$

Thus, assuming $\langle {\tilde{v}}^{2}\left(0\right)\rangle =k_{B}T$ we have

(43) $$\begin{array}{c} k_{B}\theta \left(t\right)=k_{B}T+\;\;\;\;\;\;\;\;\;\;\;\;\;\;\;\;\;\;\;\;\;\;\;\;\;\;\;\;\;\;\;\;\;\;\;\;\;\;\;\;\;\;\;\;\;\;\;\;\;\;\;\;\;\;\\ \int_{0}^{t}\int_{0}^{t}R\left(\tau \right)R\left(\tau^{\prime }\right)\langle X\left(t-\tau \right)X\left(t-\tau^{\prime }\right)\rangle d\tau d\tau^{\prime }. \end{array}$$

This result is equivalent to Equation (31), with the noteworthy aspect being its dependence on the square of the external perturbation X(t).

The temperature θ(t) enables the following energy-based rescaling:

(44) $$\frac{w^{2}\left(t\right)}{\theta (t)}=\frac{v^{2}\left(t\right)}{T},$$

where w(t) represents the rescaled fast variable. Notably, conducting the probabilistic description in terms of *w* restores the adiabatic approximation. In this sense, the probability distribution of *w* follows a Gaussian form form:

(45) $$\begin{array}{cc} f\left(w\right)={\left(\frac{1}{2\pi M(t)}\right)}^{1/2} & \\ e^{-{\left[w-w\left(0\right)R_{w}\left(t\right)-\int_{0}^{t}R_{w}\left(t-\tau \right)X\left(\tau \right)d\tau \right]}^{2}/2M\left(t\right)}, \end{array}$$

where now the variance is given by $M\left(t\right)=k_{B}\theta \left(t\right)\left[1-R^{2}\left(t\right)\right])$, and

(46) $$R_{w}\left(t\right)=\frac{\langle w(0)w(t)\rangle }{\langle w^{2}\left(0\right)\rangle }=\sqrt{\frac{\theta }{T}}R\left(t\right).$$

This Gaussian form is compatible with the velocity histograms of the tracer particle for different pulling forces and potentials corresponding to Figure 5 in Ref. [23]

On the other hand, it can be proven that

(47) $$\frac{d}{dt}w\left(t\right)=-\int_{0}^{t}\kappa_{w}\left(t-\tau \right)w\left(\tau \right)d\tau +\sqrt{\frac{T}{\theta }}\zeta \left(t\right),$$

where ζ(t) is the rescaled noise, defined by

(48) $$\frac{F(t)}{T^{1/2}}=\frac{\zeta }{\theta^{1/2}}.$$

This fulfills the fluctuation–dissipation relation

(49) $$\langle \zeta \left(t\right)\zeta \left(t^{\prime }\right)\rangle =k_{B}\sqrt{\zeta \left(t\right)\zeta \left(t^{\prime }\right)}\;\kappa \left(t-t^{\prime }\right).$$

The rescaling relation (44) is crucial for analyzing the system. It is this relation that permits the Langevin Equation (47) to be expressed in the adiabatic limit, leading directly to the noise term defined by Equations (48) and (49). Furthermore, the rescaling relation enables the derivation of the mean-field diffusion Equation (32). Consequently, any external force or perturbation modifies the system’s thermal energy. This change, in turn, alters the temporal correlations of the noise, thereby modifying the FDT. It is important to note that this external perturbation is not necessarily a systematic force. Instead, it can originate from non-thermal noise, such as that arising from the chaotic dynamics of a set of coupled variables. Throughout this framework, the definition of temperature (θ) has been treated as a fundamental postulate.

The following discussion addresses this effect in terms of the first form of the FDT, which involves response functions and time correlation functions.

### 3.3. FDT and Response Functions [17,18]

Considering the relaxational dynamics outlined in Equation (32), the presence of a fluctuation–dissipation theorem (FDT) is worth exploring. Hence, we must look for the relation between the time derivative of the correlation function $C_{AF}\left(t,t^{\prime }\right)$ with the response function $R_{AF}\left(t,t^{\prime }\right)$ of an observable A(x) through the mean-field “temperature” when, at time $t=t_{0}$, the system is perturbed by the external field F(x,t).

For a sufficiently weak perturbation around the quasi-equilibrium state characterized by Equation (34) and the mean-field “temperature” $\theta_{mf}$, the response of the system can be expressed in terms of the deviation of the average value ${\langle A\left(t\right)\rangle }_{F}$ in the presence of the force field *F* with respect to the unperturbed case ${\langle A\left(t\right)\rangle }_{qe}:$

(50) $${\langle A\left(t\right)\rangle }_{F}-{\langle A\left(t\right)\rangle }_{qe}=\int_{t_{0}}^{t}R_{AF}\left(t,t^{\prime }\right)F\left(t^{\prime }\right)dt^{\prime },$$

where we have defined

(51) $${\langle A\left(t\right)\rangle }_{F}=\int A\left(x\right)\rho_{F}\left(x,t\right)dx$$

and

(52) $${\langle A\left(t\right)\rangle }_{qe}=\int A\left(x\right)\rho_{qe}\left(x,t\right)dx$$

To obtain an explicit expression for the left-hand side of Equation (51), we may use the fact that the perturbed probability density $\rho_{F}$ satisfies the identity

(53) $$\rho_{F}\left(x,t\right)=\int_{t^{\prime }<t}\int G_{F}\left(x,t|x^{\prime },t^{\prime }\right)\rho_{F}\left(x^{\prime },t^{\prime }\right)dx^{\prime }dt^{\prime },$$

in application of the Law of Total Probability for inhomogeneous time processes. In addition, $G_{F}\left(x,t|x^{\prime },t^{\prime }\right)$ is the Green function in the presence of the perturbation.

We can similarly apply the Total Probability Law to the quasi-equilibrium solution of Equation (32) in the presence of the perturbation *F*, denoted as $\rho_{qe}^{F}\left(x,t\right)$

(54) $$\rho_{qe}^{F}\left(x,t\right)=\int_{t^{\prime }<t}\int G_{F}\left(x,t|x^{\prime },t^{\prime }\right)\rho_{qe}^{F}\left(x^{\prime },t^{\prime }\right)dx^{\prime }dt^{\prime },$$

Additionally, we can infer that $\rho_{qe}^{F}\left(x,t\right)=\rho_{qe}Z_{F}\left(x,t\right)$, where $Z_{F}\left(x,t\right)$ is a functional of the perturbation field defined as Z[F(x,t)]. Hence

(55) $$\begin{array}{cc} & \rho_{qe}\left(x,t\right)Z_{F}\left(x,t\right)=\\ & \int_{t^{\prime }<t}\int G_{F}\left(x,t|x^{\prime },t^{\prime }\right)\rho_{qe}\left(x^{\prime },t^{\prime }\right)Z_{F}\left(x^{\prime },t^{\prime }\right)dx^{\prime }dt^{\prime }, \end{array}$$

Now, after rearranging terms, one obtains

(56) $$\rho_{qe}\left(x,t\right)=\int_{t_{0}}^{t}\int G_{F}\left(x,t|x^{\prime },t^{\prime }\right)\frac{Z_{F}\left(x^{\prime },t^{\prime }\right)}{Z_{F}\left(x,t\right)}\rho_{qe}\left(x^{\prime },t^{\prime }\right)dx^{\prime }dt^{\prime }$$

Using Equation (56), and again, the Law of Total Probability applied to $\rho_{qe}\left(x,t\right)$, we may establish the following relation between Green functions

(57) $$G_{F}\left(x,t|x^{\prime },t^{\prime }\right)=\frac{Z_{F}\left(x,t\right)}{Z_{F}\left(x^{\prime },t^{\prime }\right)}G\left(x,t|x^{\prime },t^{\prime }\right)$$

which expresses the perturbed Green function in terms of the unperturbed one, $G(x,t|x^{\prime },t^{\prime })$. Now, assuming that $\rho_{F}\left(x,t_{0}\right)=\rho_{qe}\left(x,t_{0}\right)$, then the following relation holds:

(58) $$\rho_{F}\left(x,t\right)=\int_{t_{0}<t}\int G_{F}\left(x,t|x_{0},t_{0}\right)\rho_{qe}\left(x_{0},t_{0}\right)dx_{0}dt_{0}$$

The substitution of Equation (58) into Equation (51) leads, after using the fluctuation relation (57), to the formula

(59) $$\begin{array}{ccc} & {\langle A\left(t\right)\rangle }_{F}=\int_{t_{0}<t}\int \int A\left(x\right)\frac{Z_{F}\left(x,t\right)}{Z_{F}\left(x_{0},t_{0}\right)}\times & \\ & G\left(x,t|x_{0},t_{0}\right)\rho_{qe}\left(x_{0},t_{0}\right)dxdx_{0}dt_{0} \end{array}$$

We can write Equation (59) in a form similar to Equation (50) by approximating the quotient $Z\left[F\left(x,t\right)\right]/Z\left[F\left(x_{0},t_{0}\right)\right]$ to first order in *F* using a series expansion. This operation gives the integral relation

(60) $$\begin{array}{cc} & {\langle A\left(t\right)\rangle }_{F}-{\langle A\left(t\right)\rangle }_{qe}=\int_{t_{0}<t}\int \int A\left(x\right)\left[F\left(x,t\right)-F\left(x_{0},t_{0}\right)\right]\times \\ & G\left(x,t|x_{0},t_{0}\right)\rho_{qe}\left(x_{0},t_{0}\right)dxdx_{0}dt_{0} \end{array}$$

In order to take a step forward, we define the previously undefined F(x,t) as $F_{0}dB\left(x\right)/dx$, where $F_{0}$ is assumed to be a constant. Hence, now for the Smoluchowski operator *Z*, one has

(61) $$Z\left[F\left(x,t\right)\right]=\frac{\exp(F_{0}B\left(x\right)/k_{B}\theta_{mf})}{\langle \exp\left(F_{0}B\left(x\right)/k_{B}\theta_{mf}\right)\rangle }.$$

Therefore, substituting Equation (61) into Equation (60), and considering weak perturbations where $\exp\left(F_{0}B/k_{B}T\right)∼1-F_{0}B/k_{B}T$, we obtain

(62) $$\begin{array}{ccc} & {\langle A\left(t\right)\rangle }_{F}-{\langle A\left(t\right)\rangle }_{qe}= & \\ & F_{0}\left[\frac{1}{k_{B}\theta_{mf}\left(t\right)}C_{AB}\left(t,t\right)-\frac{1}{k_{B}\theta_{mf}\left(t_{0}\right)}C_{AB}\left(t,t_{0}\right)\right] \end{array}$$

where $C_{AB}\left(t,t_{0}\right)=\langle A\left(t\right)B\left(t_{0}\right)\rangle -\langle A\left(t\right)\rangle \langle B\left(t_{0}\right)\rangle$. Let us now assume that the quasi-stationary state of the system varies slowly enough, then we can replace $\theta_{mf}\left(t\right)$ by $\theta_{mf}\left(t_{0}\right)$ in Equation (62). The resulting expression can then be written as the integral of the time derivative of the correlation function which, after being compared with Equation (50), finally gives

(63) $$R_{AB}\left(t,t_{0}\right)=\frac{1}{k_{B}\theta_{mf}\left(t_{0}\right)}\frac{\partial }{\partial t_{0}}C_{AB}\left(t,t_{0}\right)$$

which makes the quasi-equilibrium fluctuation–dissipation relation valid for times $t>t_{0}$. This result implies that the fluctuation–dissipation relation can be generalized to the quasi-equilibrium state by incorporating the corrections on the system’s temperature due to the large potential gradient ∇V(x). Clearly, this result is fully compatible with the relation (57).

## 4. Application to Non-Equilibrium Systems

The thermal rescaling, detailed in Section 2 and explicitly shown in Section 3, has an intrinsic characteristic length scale that is defined by the fundamental nature of the system. This length scale may be linked to geometrical constrictions or confining forces, as well as possible stochastic forces. Nevertheless, the key finding is that the temperature correction is a function of this characteristic length scale. Furthermore, the chemical potential is also dependent on this length scale. Therefore, the correction to the temperature can be expressed either in terms of the characteristic length scale or the related chemical potential, according to Equations (4) and (12).

Following the title, this section applies our effective temperature theory to the description of certain non-equilibrium systems, providing experimental support for the theory.

### 4.1. Colloidal Suspensions [24]

The Stokes–Einstein relation (SER) is a foundational principle in the theory of Brownian motion. It relates the diffusion coefficient $D_{0}$ of a spherical particle of radius *a* to the viscosity $\eta_{0}$ of the surrounding fluid at a temperature $T_{0}$ by the equation:

(64) $$D_{0}\eta_{0}=k_{B}T_{0}/6\pi a.$$

It is a specific application of the fluctuation–dissipation theorem, which fundamentally connects the random thermal motion (fluctuation) of particles to the fluid’s friction (dissipation). This relationship holds strictly at infinite dilution, but it can be extended to finite volume fractions, defined as ϕ=Nb/V, where *N* is the number of suspended particles, $b=4\pi a^{3}/3$ is the volume of an individual particle, and *V* is the total system volume. To make this extension, one considers the viscosity and diffusion coefficients as functions of ϕ, denoted by η(ϕ) and D(ϕ), respectively, and write

(65) $$D\left(\phi \right)\eta \left(\phi \right)=\frac{k_{B}T_{0}}{6\pi a}$$

**Figure 1:**
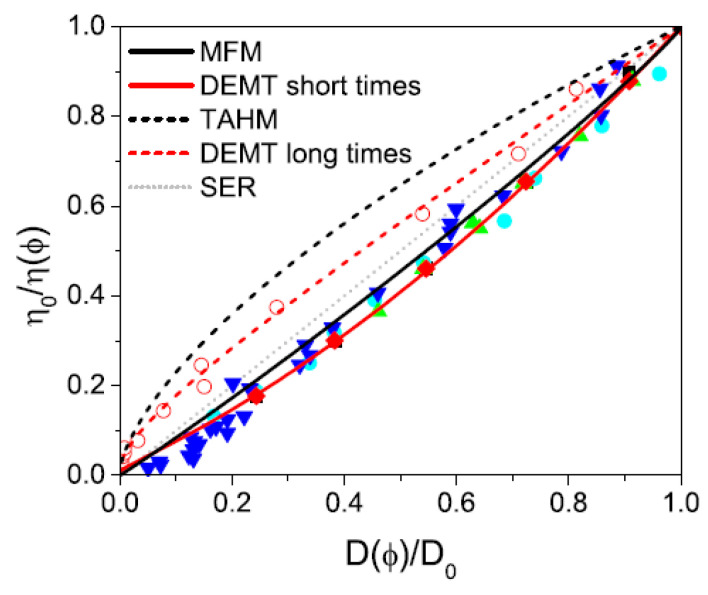
Breakdown of the Stokes–Einstein relation (SER) for short (solid lines) and long times (dashed lines). Symbols represent data from experiments and simulations taken from Refs. [25,26,27,28,29,30,31,32,33]. Here, DEMT refers to the Differential Effective Medium Theory, TAHM to the long-time Thermally Activated Hopping Model, and MFM to our Mean-Field Temperature Model. The gray-dotted straight line representing the Stokes–Einstein relation (SER) is obtained after using Equation (65) to derive the diffusion coefficient. The black solid line is the short-time mean field model (MFM) for the diffusion coefficient obtained using Equation (67). For more details, see Ref. [24]. Reproduced from [24] [dx.doi.org/10.1063/1.4930550], with the permission of AIP Publishing.

A parametric depiction of the SER, as shown in Equation (65), for colloidal suspensions, is presented in Figure 1, where the inverse of the effective viscosity (at both infinite and zero frequencies) is plotted against the diffusion coefficient (measured at short and long times, respectively).

Here, the effective viscosity is given by [34]

(66) $$\eta \left(\phi \right)=\eta_{0}{\left(1-\frac{\phi }{1-c\phi }\right)}^{-[\eta ]},$$

where [η] is the intrinsic viscosity and with c=0.22 for short times (infinite frequency) and c=0.57 for long times (zero frequency).

**Figure 2:**
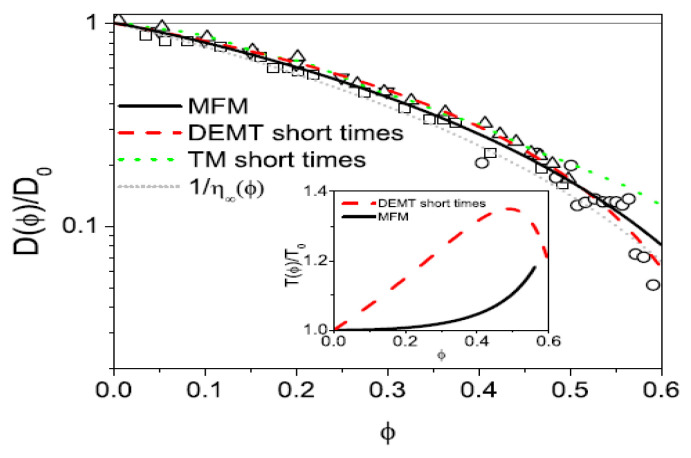
Short-time effective diffusion coefficient plotted against volume fraction. The gray dotted line represents the inverse of the infinite-frequency viscosity given by Equation (66) with c=0.22. The black solid line shows the prediction from our mean field model (MFM). The red dashed line indicates the result from the differential effective medium theory (DEMT), while the green dotted line corresponds to the prediction from the Tokuyama model (TM). The inset shows the effective temperature predicted by the indicated models. For the MFM model, T(ϕ)=θ(ϕ). Data taken from Ref. [27]. Reproduced from [24] [dx.doi.org/10.1063/1.4930550], with the permission of AIP Publishing.

At short times, the diffusion coefficient of tracer particles during their intra-cage diffusion is larger than that predicted by the SER, as evidenced by the solid lines in Figure 2. This is a consequence of the existence of strong confining forces, originated from the presence of the other suspended particles that delay the relaxation of the velocities to their equilibrium distribution. This lack of equilibration provides an extra energy supply for tracer particle fluctuations, that is, an increased thermal energy that may be quantified in terms of an “effective temperature” larger than that of the host fluid. In our framework, this effective temperature corresponds to the mean-field temperature θ(ϕ). Hence, we propose a generalized version of Equation (65), GSER, of the form

(67) $$\frac{D(\phi )}{D_{0}}\frac{\eta (\phi )}{\eta_{0}}=\frac{\theta (\phi )}{T_{0}}$$

The long-time behavior in turn, is characterized by a diffusion coefficient smaller than that predicted by the SER, as indicated by the dashed lines in Figure 1. This reduction arises because inter-cage dynamics hinder diffusion, primarily due to a sequence of thermally activated hopping events and attractive hydrodynamic interactions. To analyze this regime, the theoretical framework developed in Refs. [35,36] offers a method for calculating the long-time diffusion coefficient.

The goal now is to model the confining potential arising from the interactions between the tracer particle and the surrounding colloidal particles, which create cage-like structures that limit the tracer’s motion.

The force associated with this potential energy therefore arises from the effective heat bath, made up of the host fluid and the other colloidal particles, and depends on the particle volume fraction.

If we model the confinement using a harmonic force with a spring constant, k(ϕ), that depends on the particle concentration, the corresponding relation for the potential energy is given by

(68) $$V\left(r;\phi \right)=V_{0}+\frac{1}{2}\omega^{2}\left(\phi \right)\sum_{i=1}^{3}r_{i}^{2},$$

where $r_{i}$ are the Cartesian components of the position vector, and $\omega \left(\phi \right)=\sqrt{k(\phi )/m}$ is the corresponding characteristic frequency associated to the spring constant of the harmonic potential.

The relationship between temperature and concentration can be expressed as

(69) $$\frac{\theta (\phi )}{T_{0}}=1+\frac{1}{3}\xi^{-2}\frac{m}{k_{B}T_{0}}\epsilon^{2}\langle {\left[\nabla V\left(r;\phi \right)\right]}^{2}\rangle ,$$

and using Equation (68) we obtain

(70) $$\frac{\theta (\phi )}{T_{0}}=1+\frac{1}{3}\xi^{-2}\frac{m}{k_{B}T_{0}}\epsilon^{2}\omega^{4}\left(\phi \right)\langle r^{2}\left(t\right)\rangle$$

where $\langle r^{2}\left(t\right)\rangle$ is the mean square displacement of the tracer particles. Here, $\epsilon =\left(ma^{2}/6k_{B}T\right)\xi^{2}$ represents an Onsager coefficient that accounts for interaction effects arising from the finite size of the particles [37]. The inequality $\theta \left(\phi \right)>T_{0}$ indicates that the tracer particle possesses an excess of energy, allowing it to undergo enhanced fluctuations. This arises from the fact that the velocity distribution has not yet equilibrated.

By substituting Equation (70) into Equation (67), after calculating $\langle r^{2}\left(t\right)\rangle$ and applying Equation (66), we derive the dependence of the ratio $D\left(\phi \right)/D_{0}$ on ϕ, as illustrated in Figure 2.

### 4.2. Origin of the Effective Mobility in Nonlinear Active Micro-Rheology [19]

From the previous analysis, we can deduce that, when a constant external force, F, is applied to the particles of a suspension—resulting in a strong coupling between configurational and kinetic degrees of freedom—the corresponding effective temperature, $\theta_{mf}\left(F\right)$,

(71) $$\theta_{mf}\left(F\right)=T+\frac{m}{k_{B}\xi^{2}}{\left(\langle \nabla V(x)\rangle -F\right)}^{2},$$

incorporates the influence of this external force [18]. This effect has been observed in simulations of active nonlinear micro-rheology of supercooled liquids, as reported in Refs. [38,39], where the agreement between simulation results and theoretical predictions is remarkable.

If f(u,x,t) represents the probability density in (u,x)-space, where *x* and *u* correspond to the position and velocity of a driven particle, then the essence of the statistical description of this problem lies in the failure of the adiabatic decoupling assumption for systems in or near an arrested state, such as concentrated particle suspensions and supercooled liquids. Specifically,

(72) $$f\left(u,x,t\right)∼\phi_{eq}\left(u,t\right)\rho \left(x,t\right)$$

is *no longer valid*. Instead, we must adopt the exact formulation (18), which reveals that the time scales governing the dynamics are not well-separated. This fundamental change has deep implications for the scaling of the effective temperature and the mobility of glasses and glass-like systems.

Here, the analogue to (21) is

(73) $$J\left(x,t\right)≃-\frac{k_{B}\theta_{mf}}{m\xi }\nabla \rho \left(x,t\right)-\rho \left(x,t\right)\left[\nabla V\left(x\right)-F\right].$$

After this, the effective mobility coefficient can now be calculated as follows. First, we define the local velocity v(x,t) as

(74) J(x,t)≡ϱ(x,t)v(x,t),

such that

(75) $$\langle v\left(t\right)\rangle =\frac{1}{\xi }\int_{0}^{L}\rho \left(x,t\right)\left[\nabla V(x)-F\right]dx,$$

where the characteristic length of a cage *d*, coincides with the periodicity of the potential energy V(x)=V(x+L)

(76) $$V\left(x\right)=V_{0}\left(\cos^{2}\left[\frac{2\pi x}{L}\right]+\frac{8}{10}\sin^{2}\left[\frac{2\pi x}{L}\right]\right).$$

To a first approximation, we can use the stationary solution $\rho_{st}\left(x\right)$, obtained by setting J(x,t)=0 in Equation (73), to derive the following expression for the effective mobility β(F):

(77) $$\beta \left(F\right)=-\frac{\langle v(t)\rangle }{F}=\frac{1}{m\xi }\frac{k_{B}\theta_{mf}}{F}\left(1-e^{-FL/k_{B}\theta_{mf}}\right)/A_{L,F}.$$

The factor $A_{L,F}$ in Equation (77) is defined by

(78) $$A_{L,F}=\frac{1}{L}\int_{0}^{L}e^{-Fx/k_{B}\theta_{mf}}\int_{0}^{L}e^{\left[V\left(z\right)-V\left(z+x\right)\right]/k_{B}\theta_{mf}}dzdx.$$

**Figure 3:**
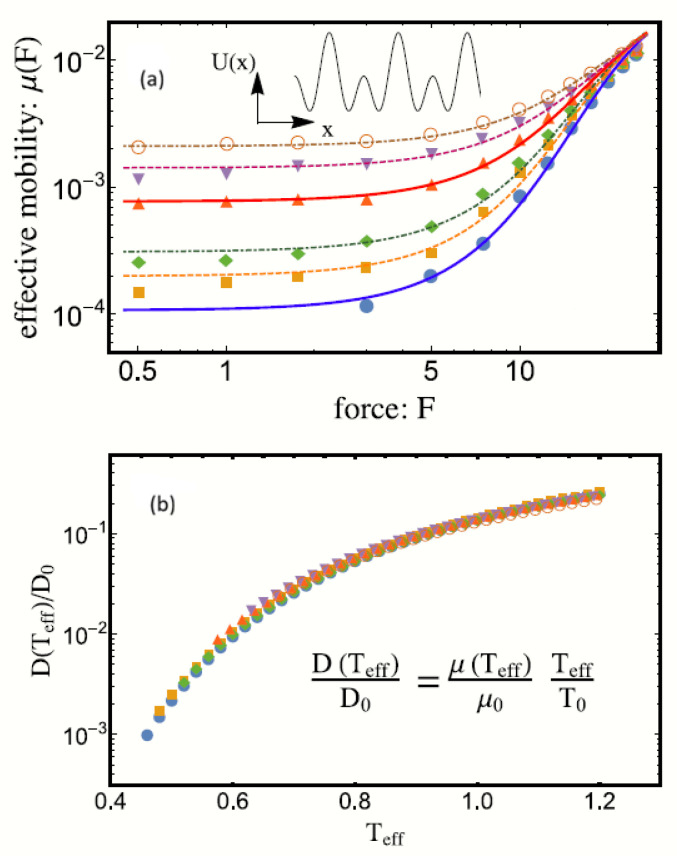
(**a**) Comparison between simulation data (symbols) taken from Ref. [40] and the results for the effective mobility given by Equation (77) (lines). (**b**) Normalized effective diffusion coefficient as a function of the effective temperature according to Equation (77). The symbol’s colors correspond to those of (**a**). Here, $T_{eff}$ should be read as $\theta_{mf}$ (defined in Equation (71)). Likewise, μ should be read as β (defined in Equation (77)). Reproduced from Ref. [19] [dx.doi.org/10.1063/1.4964283], with the permission of AIP Publishing.

By approximating the arguments of the exponentials in Equation (78) by their first-order series expansion in terms of z, it is possible to compare our results with the simulation data for the dependence of the mobility coefficient on the externally applied force [40]. As shown in Figure 3, our theoretical results exhibit excellent agreement with simulation data, validating our theory.

### 4.3. Rate Processes [20]

Perhaps one of the most important implications of the thermal scaling and the recovery of the Boltzmann statistics in Equation (13) is the reformulation of Eyring’s theory for systems far from equilibrium. The essence of Eyring’s theory of chemical reactions [35], also known as Transition State Theory (TST), is that a chemical reaction proceeds through an intermediate transition state (or activated complex) that exists at the highest energy point along the reaction pathway. One of the most significant outcomes of this theory is the formula for reaction rate constants in terms of the activation energy and partition functions.

**Figure 4:**
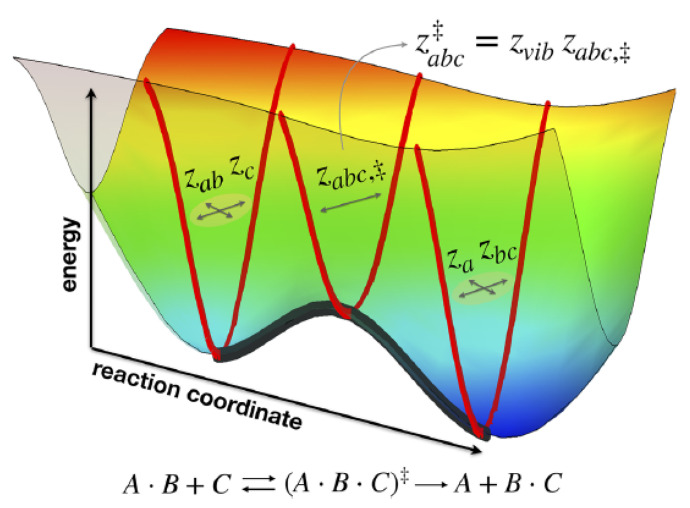
Schematic representation of the reaction path (black line) along the reaction coordinate and of the partition functions associated with the degrees of freedom orthogonal to the reaction coordinate in the reactants, activated complex, and product states (red lines). Reproduced from Ref. [20], with the permission of AIP Publishing.

The reformulation of the TST starts by assuming the existence of the quasi-equilibrium distribution function $f_{qeq}\left(\alpha ;t\right)$ for the slow variable α:

(79) $$f_{qeq}\left(\alpha ;t\right)=\frac{1}{Z[\theta (t)]}e^{-H\left(\alpha ;t\right)/k_{B}\theta \left(t\right)},$$

where $Z\left[\theta \left(t\right)\right]=\int f_{qeq}d\alpha$ is the partition function. Furthermore, the Hamiltonian $H\left(\alpha ;t\right)\equiv H_{t}$ is time-dependent due to the contribution of the force $X_{\alpha }\left(t\right)$. Likewise, in a chemical reaction occurring within a reactor at temperature θ(t), the equilibrium constant varies with temperature through its dependence on the partition functions of the reactants and the activated complex, as described by transition state theory. For example, in the case of a bimolecular reaction (illustrated in Figure 4) [35]

(80) $$A\cdot B+C\overset{K^{‡}}{⇄}\;{\left(A\cdot B\cdot C\right)}^{‡}\overset{K}{⟶}\;A+B\cdot C,$$

the equilibrium constant for the formation of the activated complex, $K^{‡}\left(\theta \right)$, which is a key component of the rate constant expression, is directly related to the partition functions of the reactants and the activated complex, is written as

(81) $$K^{‡}\left(\theta \right)=\frac{z_{abc}^{‡}}{z_{ab}z_{c}}e^{-E_{0}/k_{B}\theta },$$

where $E_{0}$ is the activation energy, $z_{i}$ are the molecular partition functions of reactants, and $z_{abc}^{‡}$ is the partition function of the activated complex. Further manipulation of Equation (81) leads to expression of the observed rate constant

(82) $$k_{obs}∼\frac{k_{B}\theta }{h}e^{\Delta G^{‡}/k_{B}\theta },$$

where $\Delta G^{‡}\left(\theta \right)$ is the Gibbs energy of activation. The previous Equation (82) constitutes the generalization of the Eyring equation for systems far away from equilibrium. Likewise, we have derived the important relation

(83) $$E_{a}=\frac{T}{\theta }\Delta G^{‡}.$$

Equivalent results for reaction rate constants can also be obtained using non-equilibrium mesoscopic thermodynamics [41,42] In order to perform this analysis, we will assume that *x* represents the reaction coordinate and that the reaction itself can be described as a diffusion process along the reaction coordinate. For chemical systems, G(x) is the free energy controlling the reaction. Following [41], the current J(x,t) given through Equation (21) can be rewritten in the mean-field form as

(84) $$J\left(x,t\right)=-D_{mf}e^{-G\left(x\right)/k_{B}\theta_{mf}}\nabla e^{\mu \left(x,t\right)/k_{B}\theta_{mf}}$$

where we have introduced the chemical potential

(85) $$\mu \left(x,t\right)=k_{B}\theta_{mf}\ln\rho \left(x,t\right)+G\left(x\right)$$

When the height of the energy barrier separating the two minima of the potential is large compared to thermal energy, a fast relaxation toward the local minima occurs. Then, the photochemical potential becomes a piece-wise continuous function of the coordinates:

(86) $$\mu \left(x,t\right)=\mu^{-}\left(x,t\right)\;\Theta \left(x_{0}-x\right)+\mu^{+}\left(x,t\right)\;\Theta \left(x-x_{0}\right)$$

with ($\mu^{-}$) and $\left(\mu^{+}\right)$ referring to the chemical potential at the left and right wells, respectively. Consequently, the probability density also splits as

(87) $$\begin{array}{cc} & \rho \left(x,t\right)=\rho_{1}\left(t\right)e^{-\left[G\left(x\right)-G\left(x_{1}\right)\right]/k_{B}\theta_{mf}}\;\Theta \left(x_{0}-x\right)+\\ & \rho_{2}\left(t\right)e^{-\left[G\left(x\right)-G\left(x_{2}\right)\right]/k_{B}\theta_{mf}}\;\Theta \left(x-x_{0}\right) \end{array}$$

here, $\left(\rho_{i}\left(t\right)=\rho \left(x_{i},t\right)\right)$ with (i=1,2), are the values of the probability density at the minima, Θ(x) is the step function, and $\left(x_{j}\right)$ with (j=0,1,2) are the coordinates of the maximum and minima of the potential, respectively.

Starting from the mean-field Smoluchowski Equation (32) and using relations (84)–(87), it is possible to derive the following kinetic equation for the concentrations $\left(\rho_{i}\right)$:

(88) $$\frac{d\rho_{1}}{dt}=-\frac{d\rho_{2}}{dt}=k_{mf}^{+}F_{2}-k_{mf}^{-}\rho_{1}$$

where the forward and backward reaction rates are given by

(89) $$k_{mf}^{+,-}=D_{mf}\sqrt{\frac{G^{\prime \prime }\left(x_{2,1}\right)}{|G^{\prime \prime }\left(x_{0}\right)|}}\frac{1}{2\pi k_{B}\theta_{mf}}\exp\left(\frac{G\left(x_{2,1}\right)-G\left(x_{0}\right)}{k_{B}\theta_{mf}}\right)$$

**Figure 5:**
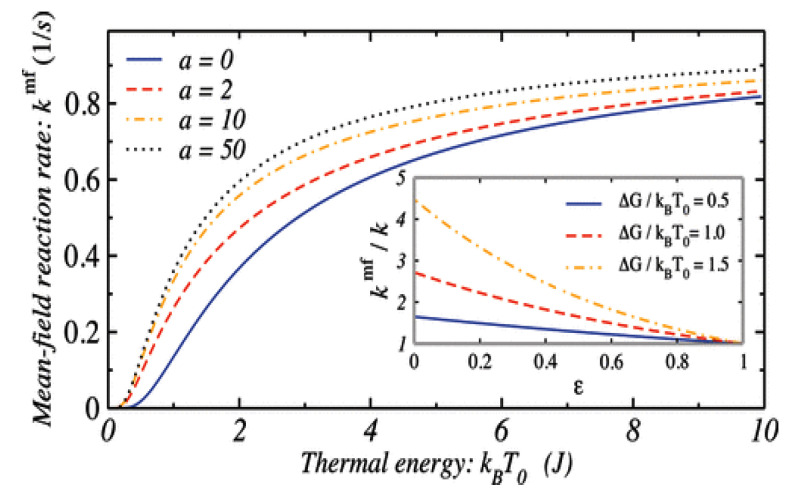
Schematic representation of the mean-field reaction rate $k_{mf}$ as a function of the temperature of the bath for different values of the correction term $a\equiv \left(1/m\right)\langle {\left(\xi^{-1}\nabla G\right)}^{2}\rangle$, with $\Delta G\equiv G\left(x_{0}\right)-G\left(x_{2,1}\right)=1$ and $D_{mf}\left(G^{\prime \prime (x_{2,1})}\right|G^{\prime \prime }$$\left(x_{0}\right){\left|\right)}^{1/2}/\left(2\pi k_{B}\theta_{mf}\right)=1$. The inset shows the ratio $k_{mf}/k$ of reaction rates as a function of $\varepsilon =T_{0}/\theta_{mf}$ for three different values of $\Delta G/k_{B}T_{0}$. When the force associated with the energy G(x) is large, the thermal energy is large enough to increase the mean-field reaction rate. Note that, in the title of the axes, $k^{mf}$ should be read as $k_{mf}$ due to technical limitations in creating a unified notation. The superscript mf is a stand-in for the intended subscript mf. Reproduced with permission from [18] [dx.doi.org/10.1021/jp204459b]. Copyright © 2011 American Chemical Society.

In Figure 5, we numerically illustrate Equation (89).

## 5. Einstein’s Absorption–Emission Theory Applied to Photovoltaics [43,44]

A case exemplifying a system far from equilibrium due to external, non-mechanical agents is the absorption and emission of electromagnetic energy. Two solar spectra with maxima at different wavelengths are observed. The difference between the Wien wavelengths (solar-plate) can be considered a characteristic length scale associated with the problem.

Here, we show how our theory, based on the effective temperature θ or the equivalent chemical potential μ, provides a procedure to describe this process.

Einstein’s theory of radiation [45,46] describes the interaction of light with a two-level atomic system by establishing a master equation. This equation governs the rates of transition between a lower energy state (*m*) with energy $E_{m}$ and a higher energy state (*n*) with energy $E_{n}$. These transitions involve the processes of absorption, spontaneous emission, and stimulated emission of photons, where the photon’s energy (hν) precisely matches the absolute energy difference between the two states ($|E_{n}-E_{m}|$). At thermal equilibrium, the populations of the *m* and *n* states, denoted by *M* and *N*, respectively, satisfies the canonical relation

(90) $$\frac{N}{M}=\frac{g_{n}}{g_{m}}\exp h\nu /k_{B}T,$$

where *T* is the equilibrium temperature.

**Figure 6:**
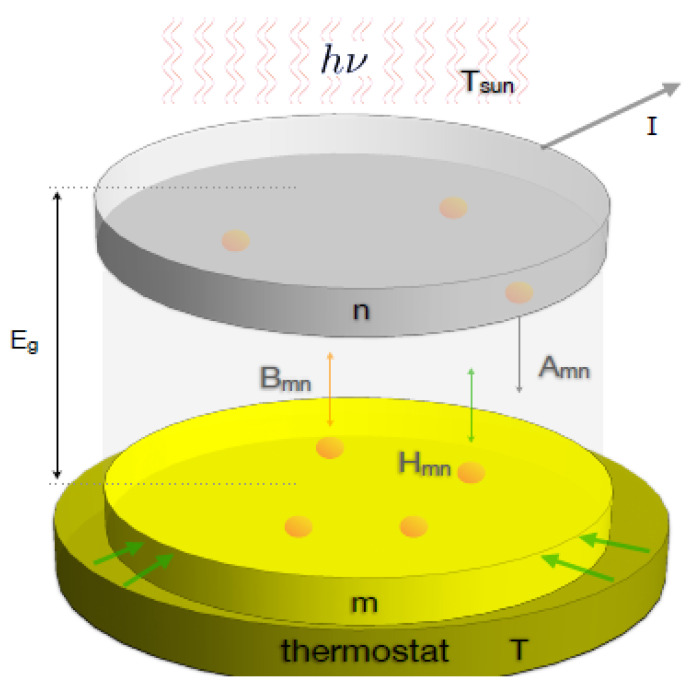
Schematic representation of the system considered. A junction in thermal contact with a thermostat at temperature T. The incoming radiation induces transitions of electrons from the valence (*m*) to the conduction (*n*) bands separated by the gap energy Eg at the rates indicated. Reproduced from Ref. [43], with the permission of MDPI Publishing.

To generalize this to non-equilibrium radiative energy exchange—typical of photovoltaic cells—we consider a semiconductor junction that exchanges energy with (i) the incoming radiation from the Sun and (ii) a thermal bath through heat conduction (see Figure 6).

The absorption of light by a semiconductor triggers photoexcitation, where incoming photons transfer energy to electrons. This energy allows electrons to transition from the valence band to the conduction band, simultaneously generating an electron–hole pair. The creation of these mobile charge carriers fundamentally alters the semiconductor’s electronic properties and enhances its electrical conductivity.

To understand this, we can often use a simplified two-level atom model. This model focuses on the essential physics, especially when we do not explicitly consider how electrons and “holes” recombine [47,48]. Even though this basic model may not explicitly detail complex recombination processes, it still accounts for electrons moving from higher to lower energy levels via radiative recombination, spontaneous emission, and thermal effects. Each of these processes is characterized by its own distinct recombination rate.

In this model, transitions occur between the semiconductor’s ground state and its excited state. These states correspond to the quasi-Fermi energy levels of the valence band ($E_{m}$) and conduction band ($E_{n}$), respectively, as described by [47]. These transitions involve the absorption and emission of photons with energy (hν) approximately equal to the semiconductor’s optical bandgap ($E_{g}$). The optical bandgap is the difference in energy between the two states, given by ($E_{n}-E_{m}$).

By generalizing Einstein’s theory of radiation, we can formulate a rate equation as follows

(91) $$\begin{array}{cc} \frac{dM}{dt} & =-\frac{dN}{dt}=-\left[B_{mn}u_{\nu }^{\mathrm{sun}}+H_{mn}\right]M\\ \; & +[B_{nm}u_{\nu }^{\mathrm{sun}}+A_{nm}+H_{nm}]N \end{array}$$

where it is essential to emphasize that, since the electromagnetic energy density producing the transitions has its origin in an external source, for example, the Sun, it approximately follows Planck’s radiation formula:

(92) $$u_{\nu }^{\mathrm{sun}}=\frac{8\pi h\nu^{3}}{c^{3}}\frac{\epsilon_{\nu }^{\mathrm{sun}}}{e^{h\nu /kT_{\mathrm{sun}}}-1}$$

where $T_{\mathrm{sun}}\approx 5250–5600\;K$. In addition, $B_{mn}$, $B_{nm}$, and $A_{nm}$ are Einstein’s coefficients [46]. Likewise, the coefficients $H_{mn}$ and $H_{nm}$ account for the thermal interaction with the thermostat, both satisfying the canonical relation, $H_{nm}/H_{mn}=\left(g_{m}/g_{n}\right)e^{h\nu /k_{B}T}$, where $g_{m}$ and $g_{n}$ represent the possible degeneracies of the states *m* and *n*, respectively.

Einstein’s theory of absorption and emission of radiation is a phenomenological theory embodied by Equation (91). Here, we added the thermostat’s interaction, not considered in the original model [46]. Hence, our approach depends on two inputs, $A_{nm}$ and $H_{mn}$. Non-relativistic quantum electrodynamics [49,50] establishes the microscopic foundations of Einstein’s phenomenological theory by providing the following expression for the spontaneous transition rate:

(93) $$A_{nm}\approx \frac{|p_{nm}{|}^{2}}{3\hbar \rho_{n}^{3}c^{3}},$$

where $p_{nm}$ is the transition dipole matrix element that governs the optical transition between the initial state *m* and the final state *n*.

Furthermore, we take advantage of the relationship between the absorption cross-section, $\alpha_{\lambda }$, and the transition dipole moment to express the Einstein B coefficient. This allows us to relate the probability per unit time of stimulated emission to that of spontaneous emission in the form:

(94) $$B\left(\lambda \right)=\frac{\lambda^{3}}{8\pi \hbar }A\left(\lambda \right),$$

where λ represents the wavelength. The relation between the spontaneous transition rate and the absorption cross-section is

(95) $$A\left(\lambda \right)=\frac{8\pi }{\lambda^{2}}\alpha_{\lambda },$$

and the corresponding relation with the stimulated probability is $B_{mn}\left(\lambda \right)=\left(\lambda /\hbar \right)\alpha_{\lambda }$ [51]. For the absorption cross section, we propose the following mathematical relation:

(96) $$\alpha \left(\lambda \right)=\frac{\lambda^{2}}{8\pi }A_{0}f\left(\lambda \right),$$

where $A_{0}$ is a characteristic transition frequency and f(λ) is the line shape function having dimensions of time.

The coefficients $H_{mn}$ and $H_{nm}$ measure the rates at which the electrons jump between the valence and conduction bands due to the thermal interaction with the heat bath. This thermal process suggests that $H_{mn}$ can follow Eyring’s formula derived from transition state theory (TST) [35]:

(97) $$H_{mn}\approx \frac{kT}{\hbar }z_{nm}\left(T\right)e^{-E_{gap}/kT},$$

where $E_{gap}$ plays the role of an activation energy and $z_{nm}\left(T\right)=z_{n}/z_{m}$ with $z_{n}$ and $z_{m}$ the partition functions of the excited and basal states, respectively. For the sake of simplicity, in the following, this rate will be considered as a constant.

The temperature difference between the Sun and the semiconductor acts as a drift that takes the semiconductor’s state away from equilibrium. Hence, it is convenient to rewrite Equation (91) in terms of the difference between electromagnetic energy densities: $\left(u_{\nu }^{\mathrm{sun}}-e_{\nu }\right)$, where $e_{\nu }$ represents the spectral energy density of the semiconductor. Thus, by simple algebraic manipulations, adding and substracting terms, we obtain

(98) $$\frac{dM}{dt}=-\frac{dN}{dt}=-j-k_{+}M+k_{-}N,$$

where

(99) $$j\equiv -B_{mn}\left(u_{\nu }^{\mathrm{sun}}-e_{\nu }\right)\left(N-M\right)$$

is the net radiative current and $k_{+}$, $k_{-}$ are reaction constants given by

(100) $$\begin{array}{cc} k_{+} & \equiv B_{mn}e_{\nu }+H_{mn},\\ k_{-} & \equiv \left(g_{m}/g_{n}\right)B_{mn}e_{\nu }+A_{nm}+H_{nm}. \end{array}$$

The degeneracy factors $g_{m}$ and $g_{n}$ enter Equation (100) because the assumption of detailed balance, $g_{m}B_{mn}=g_{n}B_{nm}$.

According to Equation (99), *j* is proportional to the net radiation received by the semiconductor from the Sun, which promotes the transference of atoms from the ground to their excited state.

In the stationary state, a non-zero non-equilibrium current,

(101) $$j_{st}=k_{+}N_{st}-k_{+}M_{st},$$

or equivalently

(102) $$j_{st}=-B_{mn}\left(u_{\nu }^{\mathrm{sun}}-e_{\nu }\right)\left(N_{st}-M_{st}\right),$$

indicates that the stationary populations, $M_{st}$ and $N_{st}$ do not satisfy the detailed balance condition. These populations are the stationary solutions of Equation (98) (when the time derivatives vanish). Nevertheless, by introducing a temperature θ (the one discussed above), we can restore a canonical-like relation, treating these populations as if they were in thermal equilibrium with an effective bath at temperature θ:

(103) $$\frac{N_{st}}{M_{st}}=\frac{g_{n}}{g_{m}}e^{-h\nu /k_{B}\theta }.$$

Here, we emphasize that θ is a function of the stationary current $j_{st}$, which can be determined using the normalization condition $N_{st}+M_{st}=1$ and Equation (101). Alternatively, as in Equation (12), θ can be replaced by a chemical potential, or more properly, a photochemical potential μ as

(104) $$\frac{N_{st}}{M_{st}}=\frac{g_{n}}{g_{m}}e^{-h\nu /k_{B}T}e^{\mu /k_{B}T}.$$

This potential measures how far the system is from equilibrium. The expression of the photochemical potential, μ, can be obtained with the aid of the normalization condition and Equations (102) and (104), giving

(105) $$\mu =k_{B}T\ln\left|\frac{1+\frac{B_{mn}}{k_{+}}\Delta u_{\nu }}{1+\frac{B_{mn}}{k_{-}}\Delta u_{\nu }}\right|,$$

where $\Delta u_{\nu }=u_{\nu }^{sun}-e_{\nu }$. A straightforward algebraic manipulation yields a more insightful expression for μ:

(106) $$\mu =k_{B}T\ln\left|1+\frac{B_{mn}\left(\frac{1}{k_{+}}-\frac{1}{k_{-}}\right)\Delta u_{\nu }}{1+\frac{B_{mn}}{k_{-}}\Delta u_{\nu }}\right|.$$

The last two formulas show that the photochemical potential is determined by the difference in electromagnetic energy density between incoming sunlight and the energy that the semiconductor radiates. This difference ($\Delta u_{\nu }=u_{\nu }^{sun}-e_{\nu }$) induces a spectral drift at the semiconductor’s surface, which in turn disrupts the internal energy balance of the material across various frequencies and wavelengths. If the photochemical potential is strong enough to overcome the junction’s potential barrier, this unbalance generates an electrical current.

### 5.1. Spectral Response, Quantum Efficiency, and Photochemical Potential

The main contributions of our statistical mechanical approach to photovoltaic cells are the non-equilibrium current relationship in Equation (99) and the equivalent chemical potential formulations (Equations (105) and (106)). These equations offer crucial insights into the junction’s electrical current by explicitly quantifying the impact of the drift $\Delta u_{\nu }$ on the junction’s electrical current, thereby illustrating the concepts discussed above.

#### 5.1.1. The Current–Voltage Equation

After accounting for radiative, spontaneous, and thermal recombination processes, Equation (99) represents the net flow of atoms per unit time moving from the valence band to the conduction band. This means that Equation (99) also defines the net electric current produced by the illuminated junction. The light-generated current under short-circuit conditions can be defined as

(107) $$I_{n}=\frac{qj_{st}}{A},$$

where *q* is the elementary charge and *A* is the conduction cross-section area. Using the right-hand side of Equation (99), the light-generated current can be expressed as

(108) $$I_{n}=\frac{q}{A}k_{+}M_{st}\left(\frac{k_{-}N_{st}}{k_{+}M_{st}}-1\right).$$

At equilibrium, the current density (*j*) is zero. From this, and with a reference to Equation (98), we can see that the ratio of the steady-state concentrations, $N_{st}/M_{st}$, approaches the value predicted by the Boltzmann distribution:

(109) $$\frac{N_{st}}{M_{st}}\to \frac{N_{eq}}{M_{eq}}=\frac{k_{+}}{k_{-}}=\frac{g_{n}}{g_{m}}e^{-h\nu /k_{B}T},$$

indicating that in this situation θ→T. Hence, taking into account the previous relation and in view of Equation (104), the light-generated current can be rewritten in the more compact form:

(110) $$I_{n}=I_{0}\left(e^{\mu /k_{B}T}-1\right),$$

where $I_{0}\equiv qk_{+}M_{st}/A$ and where the equation explicitly shows the dependence of the light-generated current on the photochemical potential μ. The term $I_{0}$ represents the characteristic current, which is determined by the combined production of free electrons due to both light and thermal energy. This equation is analogous to the classical short-circuit current voltage [48]

(111) $$I_{sc}={\tilde{I}}_{0}\left(e^{qV_{oc}/k_{B}T}-1\right).$$

The resemblance between Equations (110) and (111) leads us to conclude that the photochemical potential is equivalent to the voltage produced by the p-n junction

(112) $$\mu =qV_{oc}.$$

In these equations, $I_{sc}$ represents the short-circuit current and ${\tilde{I}}_{0}$ is the reverse saturation current.

The preceding analysis provides a way to express key spectral characteristics, giving precise information about the performance of p-n junctions and photovoltaic cells (PVCs) as a function of the photochemical potential.

The first result that follows from the previous analysis is the explicit expression of the spectral response $S_{r}$. This quantity is the light-generated current by the cell, $I_{n}$, divided by the power input of the incident light, $P_{i}$:

(113) $$S_{r}=\frac{I_{n}}{P_{i}}$$

In a first approximation, the short-circuit current per photon is proportional to the open cell voltage. This proportionality can be established by appealing to the Ohm’s law. Thus, from Equation (112), we obtain

(114) $$I_{n}=\frac{L}{\rho }\frac{\mu }{q},$$

where ρ, measured in Ω× meter, is the intrinsic resistivity of the semiconductor and *L* is a characteristic length of the transport process, like the minority carrier diffusion length. The input power per photon by the incident light can be written as

(115) $$P_{i}=h\nu \frac{c}{4L}$$

Therefore, using Equations (114) and (115), the spectral response becomes

(116) $$S_{r}\left(\lambda \right)=\frac{4L^{2}}{c\rho q}\frac{\lambda }{hc}\mu_{\lambda }$$

To adhere to the standard convention for this phenomenon, the representation was changed from frequency to wavelength (λ).

Since it is known that spectral response and the quantum efficiency, $Q_{e}\left(\lambda \right)$ are related through

(117) $$S_{r}\left(\lambda \right)=\frac{q\lambda }{hc}Q_{e}\left(\lambda \right),$$

the quantum efficiency is therefore given by

(118) $$Q_{e}\left(\lambda \right)=\frac{4L^{2}}{c\rho q}\mu_{\lambda }$$

**Figure 7:**
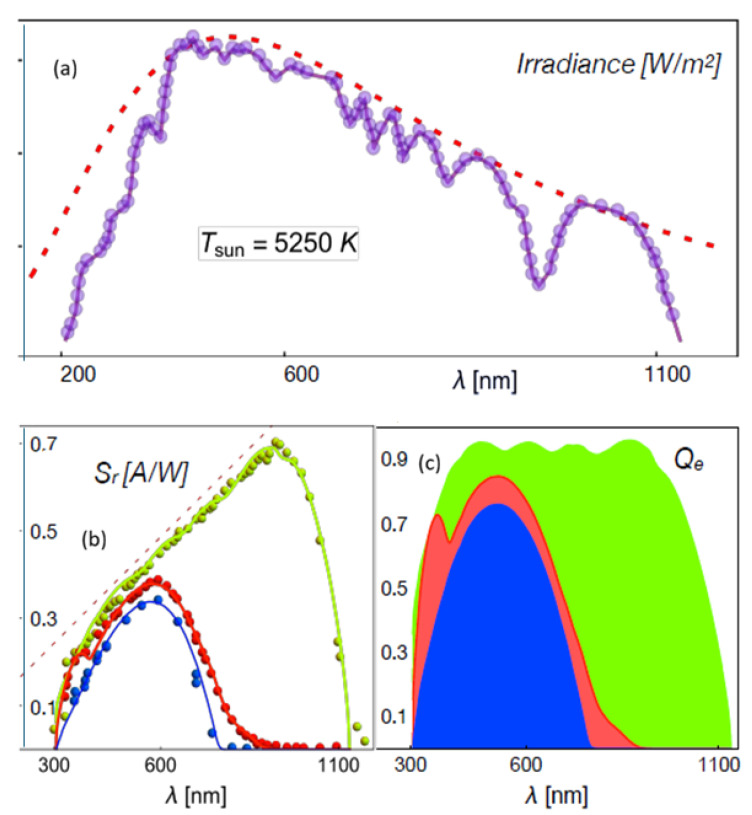
(**a**) Solar irradiance and interpolation of the data. (**b**) Spectral response, Equation (116) and (**c**) quantum efficiency, Equation (118), of different crystalline and amorphous Si-based cells [52] as a function of the wavelength for 1.5AMG. The green symbols and line correspond to the spectral response to AQ81/cr-Si, the red symbols and line correspond to AQ82/cr-Si filtered, and the blue symbols and line to AQ83(4)/a-Si. Reproduced from Ref. [43].

**Figure 8:**
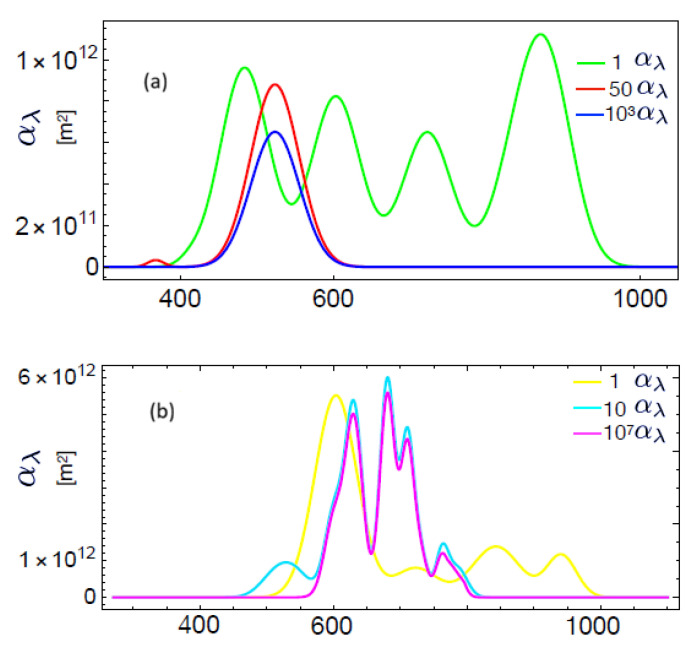
Absorption cross-section given by Equation (96) as a function of the wavelength. (**a**) The three coefficients used for the fits of Figure 7 with the same color key. (**b**) The three coefficients used for the fits of Figure 9 with the same color key. Reproduced from Ref. [43].

Thus, Equations (116) and (118) emerge as fundamental spectral quantities providing precise information about the performance of p-n junctions and, in general, of PVCs. Here, Equation (118) indicates that the photochemical potential accounting for the unbalance induced by the light input power on the p-n junction is directly associated with the quantum efficiency of the cell, a spectral quantity. We have ploted theses spectral quantities in Figure 7 which shows the data (symbols) of the spectral response of crystalline and amorphous Si-based cells reported in [52], as well as the fit (lines) using Equation (116) with the corresponding absorption cross-section shown in Figure 8. The absorption cross-sections used were modeled, in a first approximation, by adding Gaussian functions with different amplitudes and variances.

#### 5.1.2. Entropy Production

We evaluate the spectral entropy production of the semiconductor junction, which arises from three distinct operational processes: current production, light absorption, and heat production. According to the Gibbs entropy postulate [55], the total entropy of the cell depends on the populations *N* and *M* as follows:

(119) $$S_{\mathrm{cell}}=-kM\ln\left(\frac{M}{M_{\mathrm{eq}}}\right)-kN\ln\left(\frac{N}{N_{\mathrm{eq}}}\right)+S_{\mathrm{eq}},$$

where $S_{\mathrm{eq}}$ is the equilibrium entropy of the system and M(t) and N(t) evolve in time according to Equation (98). The time derivative of Equation (119) gives the total change of the entropy due to the three previously mentioned processes, since the evolution equation of the populations incorporates the corresponding energy (and entropy) exchanges due to the presence of the radiative and thermal transition rates. Thus, performing the time derivative of Equation (119), using the fact that dM/dt=−dN/dt due to number conservation, and the condition of detailed balance at equilibrium, one can write the following expression for the entropy change per unit of time:

(120) $$\frac{dS_{\mathrm{cell}}}{dt}=kj\ln\left(\frac{k_{+}M}{k_{-}N}\right)+\left(k_{+}M-k_{-}N\right)\ln\left(\frac{k_{+}M}{k_{-}N}\right).$$

The first term on the right-hand side of the last equation is the entropy exchange per unit of time (${\dot{\Phi }}_{\mathrm{cell}}$) between the cell and the surroundings. This is due to the power input associated with the absorbed incoming light, which is proportional to *j*. The exchange of heat between the cell and the thermostat enters through the forward and backward rates $k_{+}$ and $k_{-}$. Recall that these rates depend on the thermal rates $H_{mn}$ and $H_{nm}$. The second term on the right-hand side is the spectral entropy production per unit time (${\dot{\Sigma }}_{\mathrm{cell}}$) associated with the generation of free electrons due to the absorption of light and the heat exchange with the thermostat. As is well known, in the stationary state, the entropy exchanged with the surroundings compensates the entropy produced (${\dot{\Sigma }}_{\mathrm{cell}}=-{\dot{\Phi }}_{\mathrm{cell}}$) in such a way that the total entropy of the cell remains constant, $dS_{\mathrm{cell}}/dt=0$. This condition implies Equation (101) and shows the consistency of our analysis. Therefore, the entropy production per unit time, ${\dot{\Sigma }}_{\mathrm{cell}}$, can be written in the form:

(121) $${\dot{\Sigma }}_{\mathrm{cell}}=-kj\ln\left(\frac{k_{+}M}{k_{-}N}\right).$$

Using Equations (104) and (109) in order to rearrange the terms in the logarithm, it is possible to find the corresponding stationary state version of Equation (121):

(122) $${\left({\dot{\Sigma }}_{\mathrm{cell}}\right)}_{st}=\frac{j_{st}\mu_{\lambda }}{T},$$

which, in view of Equations (107) and (112), this leads to the expression for the stationary specific entropy production, ${\dot{\sigma }}_{st}\equiv {\left({\dot{\Sigma }}_{\mathrm{cell}}\right)}_{st}/A$, such that

(123) $${\dot{\sigma }}_{st}=\frac{I_{n}V_{oc}}{T}.$$

This equation notably recovers the Joule heat effect, quantifying the power dissipated due to the electric current.

## 6. Quantum Confinement

Understanding the energy-related properties of nanoscale materials and structures under quantum confinement is vital for predicting their electronic, optical, and thermal behavior. Quantum confinement occurs when material dimensions become comparable to the de Broglie wavelength of electrons (typically a few nanometers). At this scale, electrons are effectively trapped, leading to the quantization of energy levels, much like a particle in a potential well. This quantization is the key factor driving the unique electronic and optical properties observed in structures like quantum dots, nanowires, and thin films. Furthermore, quantum confinement impacts thermal properties by altering the behavior of confined phonons and their interactions with electrons, which, in turn, influences heat conduction and thermal management. These consequences of quantum confinement are central to contemporary scientific advancements.

Notably, the nanometric dimensions of quantum-confined systems significantly impact their vibrational density of states, rendering the conventional Debye model inadequate [56]. However, we demonstrate that the Debye density can be reinstated by employing a rescaled frequency that appropriately accounts for these size-dependent effects. This recovery is enabled by our novel insight: the inherent invariance of statistical mechanics under the simultaneous scaling of both energy and temperature.

Earlier, we defined two energy scales: $\hat{E}$, which now represents the bulk system’s energy at a rescaled temperature θ, and *E*, the directly observed energy of the physical system. Interestingly, the difference between both energies gives us an energy excess, ΔE, which parallels the role of the $m^{*}\mu$ term we saw with the photochemical potential, namely

(124) $$\frac{\hat{E}}{k_{B}\theta }=\frac{\hat{E}-\Delta E}{k_{B}T},$$

which, in view of Equation (10), yields the relation

(125) $$\hat{E}-\Delta E=E.$$

Therefore, rescaling is akin to introducing an energy excess ΔE. This formula establishes that *E* is the energy that contains the corrections imposed by the confinement through this energy excess.

When Equation (125) is reinterpreted in terms of frequencies as

(126) $$h\hat{\nu }-m^{*}\mu =h\nu ,$$

it is evident that confinement causes a shift in the frequencies. Here, the photophotochemical potential, μ, is interpreted as the energy excess per photon. Shifting frequencies reconfigures the entire energy structure of a material, altering its optoelectronic, thermal, and mechanical characteristics by either increasing or decreasing the energies of specific states that particles can occupy. Thus, the energy structure of a material is strongly influenced by its nanoscale size compared to its bulk form.

In cases of light emission and absorption involving nanostructures, Wien’s displacement law helps determine the effective temperature

(127) $$\theta =\frac{C_{W}}{d}.$$

Here, $C_{W}$ is the Wien’s constant and *d* is a characteristic nanoscopic length.

Under thermal rescaling, Planck’s law for the density of thermal radiation remains unchanged. Consequently, the rescaled form of Planck’s law is given by

(128) $$\hat{u}\left(\hat{\nu }\right)=\frac{8\pi {\hat{\nu }}^{2}}{c^{3}}\frac{h\hat{\nu }}{\exp\left(\frac{h\hat{\nu }}{k_{B}\theta }\right)-1},$$

where the factor $\frac{8\pi {\hat{\nu }}^{2}}{c^{3}}$ corresponds to the Debye spectral density. In this context, Equation (128) represents the spectral energy density of a blackbody (BB) at a given temperature θ. By using Equation (126), we can rewrite Equation (128) in terms of the photophotochemical potential μ

(129) $$\hat{u}\left(\hat{\nu }\right)=\frac{8\pi {\hat{\nu }}^{2}}{c^{3}}\frac{h\hat{\nu }}{\exp\left(\frac{h\hat{\nu }-m^{*}\mu }{k_{B}T}\right)-1}$$

which, as known in the literature although in a different context, represents the generalized Planck’s law [57,58,59,60,61,62].

Now, turning to Equation (11), this equation becomes even more intriguing when applied to photons in a cavity:

(130) $$E=h\hat{\nu }\frac{T}{\theta }=h\hat{\nu }\frac{T}{C_{W}}d$$

revealing that the energy per photon is adjusted based on the confinement length. The potential of the previous equation lies in the fact that it allows us to calculate the work ΔF required to confine the radiation in a cavity of volume *V* at a temperature *T* as follows

(131) $$\Delta F=\frac{VT}{\theta }\int_{0}^{\infty }\hat{u}\left(\hat{\nu }\right)d\hat{\nu }.$$

Here, using Equation (131), we can derive the pressure as an interesting result [62]:

(132) $$p=-{\left(\partial F/\partial V\right)}_{T}∼\frac{k_{B}T}{d^{3}}$$

which constitutes the thermal correction to the Casimir effect, where *V* is assumed to be the volume of a nanocavity with a constant cross-section and variable width *d*. To simplify the presentation, we have omitted the prefactors.

**Figure 10:**
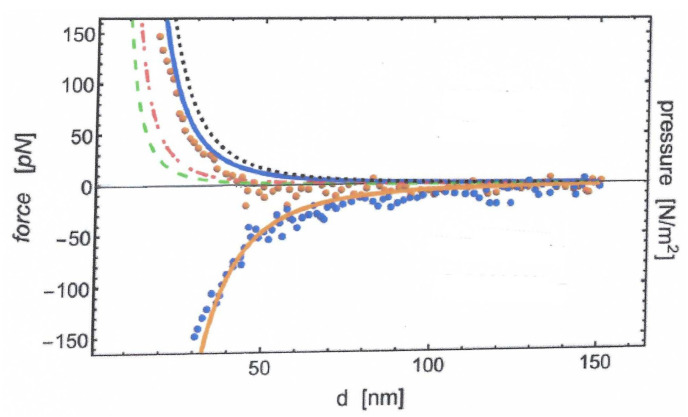
Attractive and repulsive Casimir forces. Comparison between experiments and theory. The experimental results were digitalized from Ref. [63]. The comparison between experimental data (orange symbols) and the theoretical repulsive force (blue solid line) at T=300 K is given by Equation (132) without fitting parameters. The black dotted line corresponds to the prediction from Ref. [64], the red dash-dotted line to the prediction from Ref. [65], and the green dashed line to the prediction from Ref. [66], all of them for the Casimir pressure and without fitting parameters. The attractive force, experimental (blue circles), and theory (orange solid line) is again given by Equation (132) multiplied by −4.0. Reproduced from Ref. [62] [DOI: 10.1039/d1nh00496d], with permission from the Royal Society of Chemistry.

In Figure 10, we plot the prediction of Equation (132) for the Casimir pressure in conjunction with the experimental results reported in Ref. [63] for the Casimir force in terms of the surfaces separation.

## 7. Radiative Heat Transfer in the Near-Field [62]

Our results from the previous section can be applied to a non-equilibrium phenomenon: the heat exchange between two flat plates at different temperatures, $T_{1}<T_{2}$, which are separated by a nanometric distance [62]. The energy current between these plates can be defined as

(133) $$\dot{Q}=\frac{{\left(\Delta F\right)}_{2}-{\left(\Delta F\right)}_{1}}{\tau }.$$

Here, τ is a timescale which, based on an educated guess informed by quantum principles, we infer should behave as the inverse of the plasma frequency $\omega_{p}$

(134) $$\tau ∼\frac{1}{N_{a}\omega_{p}},$$

where $N_{a}$ is the Avogadro’s number. Using Equation (131) and expanding Equation (133) to first order in the temperature difference $\Delta T=T_{2}-T_{1}$ gives

(135) $${\dot{Q}}_{d}=-G\Delta T,$$

where

(136) $$G∼\frac{\tau^{-1}}{d^{2}},$$

is the thermal conductance, with the $d^{-2}$ dependence commonly observed in experiments. The prefactors have also been omitted for simplicity.

However, unlike metals, for which the previous relation holds at any distance, this is not true for insulators. According to band theory, insulating materials can behave as conductors at very high temperatures. Within our approach, high temperatures θ correspond to very short distances. Henceforth, the frequency density must be adjusted by a form function f(d) such that

(137) $$\rho \left(\hat{\nu }\right)=\rho_{D}\left(\hat{\nu }\right)f\left(d\right),$$

where $\rho_{D}\left(\hat{\nu }\right)$ is the Debye spectral density and

(138) $$f\left(d\right)=1+\frac{d}{d_{c}}$$

is the expression of the form function to first order in the parameter $d/d_{c}$, with $d_{c}$ being a crossover distance. Consequently, the thermal conductance must be adjusted by f(d) so that

(139) $$G=G_{m}f\left(d\right),$$

where $G_{m}$ is given by Equation (136). This is how the crossover behavior is obtained, from 1/d scaling for large gaps to $1/d^{2}$ scaling for very narrow gaps, as observed in the experiments [62].

**Figure 11:**
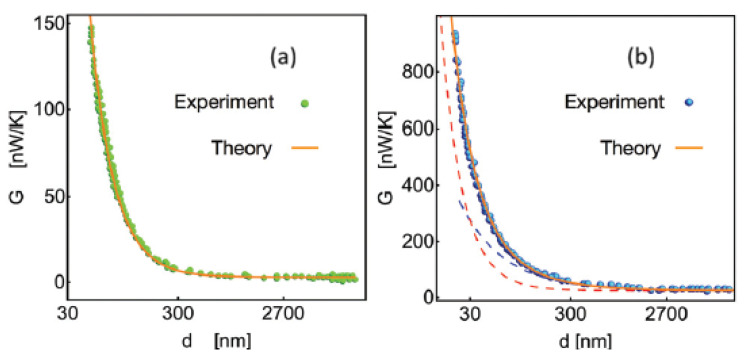
Comparison between experiment (symbols) and theory (solid orange line). (**a**) Au plates, data taken from Ref. [67] and Equation (136) with $G_{\infty }=2.5\;{\mathrm{nWk}}^{-1}$ and $\tau =1.36\times 10^{-38}$ s. (**b**) SiO_2_ surfaces, data taken from Ref. [67] and Equation (139) with $G_{\infty }=25\;{\mathrm{nWk}}^{-1}$, $\tau =2.46\times 10^{-38}$ s and $d_{c}=30$ nm. Reproduced from Ref. [62] [DOI: 10.1039/d1nh00496d], with permission from the Royal Society of Chemistry.

In Figure 11, we plot the curves representing the thermal conductance as predicted by Equations (136) and (139), alongside experimental results extracted from the literature (see Ref. [67]).

## 8. Radiative Heat Transfer in the Far-Field [68]

Building upon the results established in Section 6 and Section 7, we now turn our attention to their application in far-field radiative heat transfer, where the bodies can be separated by macroscopic distances.

In the far-field case [68], the length scale introduced by the Wien’s law is determined by the physical dimensions of the body itself. Hence, to address the issue of energy exchange in the far-field, we begin by considering the irradiance of a nanocrystal.

The energy irradiated I(ν), per frequency band, by a small body is given by the following relation $d_{\nu }I=c\alpha \left(\nu \right)u\left(\nu \right)d\nu ,$ where *c* is the speed of light in vacuum and α(ν) is the absorption cross-Section [69]. In the case of a nanocrystal of volume *V*, the previous relation translates to the following

(140) $$\begin{array}{c} d_{\nu }I=\frac{1}{\tau }V\frac{T}{\theta }\hat{u}\left(\hat{\nu }\right)d\hat{\nu }. \end{array}$$

After multiplying and dividing by a factor of 4c, Equation (140) can be compared to the well-known spectroscopic expression [70] for the absorption cross-section, allowing us to identify

(141) $$\hat{\alpha }\left(\hat{\nu }\right)=\frac{4}{\tau }\frac{V}{c}\frac{T}{\theta }.$$

Similarly, in molecular spectroscopy, it is understood that the absorption cross-section depends on the frequency through the spectral lineshape $g(\hat{\nu })$. Thus, unlike the case of near-field radiation in a vacuum cavity, we must define the characteristic time-scale here as $\tau^{-1}=\hat{\nu }g\left(\hat{\nu }\right)$.

As a result of the dependence of the optoelectronical properties on temperature, we assume that

(142) $$g\left(\hat{\nu }\right)=f\left(T\right)\tilde{g}\left(\hat{\nu }\right),$$

where f(T) can be roughly described by the maximum contribution of the Doppler effect

(143) $$f\left(T\right)∼{\left(\frac{m_{eff}c^{2}}{2k_{B}T{\hat{\nu }}_{res}^{2}}\right)}^{1/2}.$$

Here, $m_{eff}$ is an effective mass, and ${\hat{\nu }}_{res}$ is the resonant frequency.

On the other hand, in a classical approach, it is assumed that the spectral lineshape takes the form of the imaginary part of the Cole–Cole susceptibility function [70,71]

(144) $$\tilde{g}\left(\hat{\nu }\right)=\frac{{\left(\tau_{res}\hat{\nu }\right)}^{\delta }\sin\left(\pi \delta /2\right)}{1+2{\left(\tau_{res}\hat{\nu }\right)}^{\delta }\cos\left(\pi \delta /2\right)+{\left(\tau_{res}\hat{\nu }\right)}^{2\delta }}.$$

Here, δ is the characteristic exponent of the relaxation process and $\tau_{res}$ is the time at which the maximum of the absorption function occurs. Additionally, notice that, when δ<1, we have the classical response behavior associated with the Cole-Cole function [71]. However, for δ>1, the response becomes enhanced.

With this baggage, for T/θ<1 and adopting $\tau_{res}^{-1}≃k_{B}T/h$ as the physically meaningful simple assumption, we achieve [68]

(145) $$\begin{array}{c} \frac{I_{\delta }\left(T,l\right)}{\sigma T^{4}}=B_{\delta }\frac{k_{B}T}{hc}\sqrt{\frac{1}{T^{3}}}\;\sin\left(\frac{\pi \delta }{2}\right){\left(\frac{C_{W}}{Tl}\right)}^{4-\delta }V, \end{array}$$

where σ represents the Stefan–Boltzmann constant, and for the sake of notational simplicity, we have defined the constant $B_{\delta }$ by

(146) $$B_{\delta }\equiv \frac{60}{\pi^{4}}\Gamma \left(5-\delta \right)\zeta \left(5-\delta \right)\phi .$$

Here, ζ(x) is the Riemman’s zeta function and where $\phi ={\left[m_{eff}c^{2}h^{2}/\left(2k_{B}^{3}\right)\right]}^{1/2}$ has been introduced to simplify the notation.

Thus, with our result given by Equation (145) and the definition in Equation (146) at hand, we can determine the heat conductance between two anisotropic bodies at different temperatures ($T_{1}<T_{2}$), a quantity that can be experimentally verified [68]. As in the near-field case, we computed the heat current $\dot{Q}$, and from this, the heat conductance $G_{\delta }\left(l\right)$ including cooperative effects

(147) $$G_{\delta }=4F_{1,2}\frac{V}{l}\left[1+\sin\left(\frac{\pi \delta }{2}\right)\frac{k_{B}B_{\delta }}{hc}\frac{1}{T^{\frac{1}{2}}}\frac{{\left(\lambda_{T}\right)}^{4-\delta }}{l^{3-\delta }}\right]\sigma T^{3}.$$

The corresponding BB-limit of the heat conductance, $G_{bb}$, is given by

(148) $$G_{bb}=4F_{1,2}\sigma T^{3}.$$

In this context, it is essential to adhere to the established conventions in the field to introduce the view factor $F_{1,2}\left(l/d\right)$[72], a parameter that depends on the ratio of the characteristic length to the separation between the bodies, *d*, where $d>>\lambda_{T}$, where $\lambda_{T}$ is the Wien’s length at temperature *T*.

**Figure 12:**
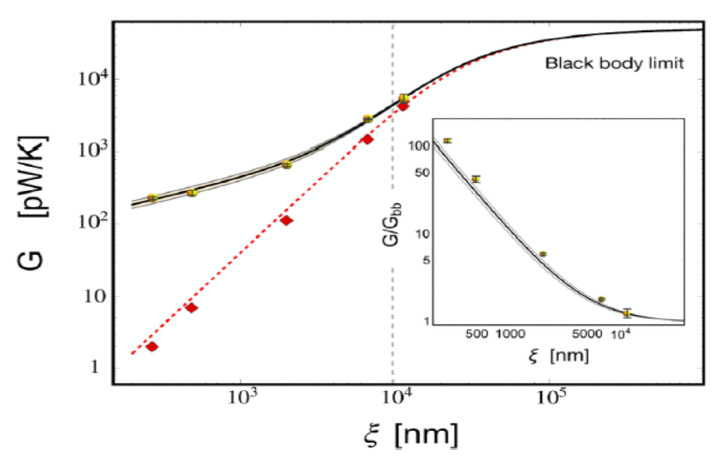
Heat exchange between two pads of dimensions $l_{x}=60\;\mu$m, $l_{y}=60\;\mu$m, and variable thickness ξ. The red squares (BB) and yellow circles (no-BB) are the experimental results from Ref. [73]. The solid black line comes from Equation (147). The dotted red line corresponds to Equation (148). The fits were done using T =300 K, $\lambda_{T}=9.8\;\mu$m, D=0.133 nm, δ=1.5, and $m_{eff}=3.63\times 10^{-12}$ kg (black line). The upper gray line corresponds to $m_{eff}=4.76\times 10^{-12}$ kg, whereas the lower gray line corresponds to $m_{eff}=2.80\times 10^{-12}$ kg. The inset shows the ratio $G_{\delta }/G_{bb}$. Details in the text. Reproduced from Ref. [68] [doi: 10.1063/5.0108448], with permission of AIP Publishing.

**Figure 13:**
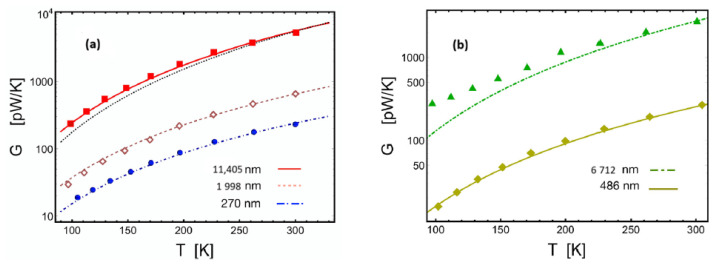
Heat conductance as a function of the temperature for different thicknesses. Symbols are experimental data taken from [73] and lines correspond to Equation (147). (**a**) Data for ξ= 270 nm (blue), 1998 nm (pink) and 11,405 nm (red). (**b**) Data for ξ= 486 nm (dark yellow) and 6700 nm (green). All fits were done by multiplying the formulas by an amplitude factor of $1.75\times 10^{-8}$. Reproduced from Ref. [68] [doi: 10.1063/5.0108448], with permission of AIP Publishing.

In Figure 12 and Figure 13, we plot the curves representing the thermal conductance as predicted by Equations (147) and (148), alongside experimental results extracted from the literature (see Ref. [73]).

## 9. Temperature Dependence of the Bandgap in Nanoscale Semiconductors [74]

In this section, we illustrate how the previously introduced theory can be utilized to explain the optoelectronic properties of nanomaterials [74]. Specifically, we focus on the unusual temperature dependence of the optical bandgap energy, which, in contrast to bulk systems, increases with temperature or exhibits a non-monotonic behavior characterized by a blueshift-redshift pattern.

The optical bandgap energy can be determined from photoluminescence experiments by identifying the peak position in the photoluminescence spectrum. Following our approach, this corresponds to the energy difference between two states in the conduction and valence bands $\Delta E_{g}=E_{c}-E_{v}\equiv h\nu_{g}$

(149) $$\Delta E_{g}=\frac{T}{\theta }h{\hat{\nu }}_{g}=\frac{T}{\theta }\frac{hc}{d_{p}},$$

where we have used the relation ${\hat{\nu }}_{g}=c/d_{p}$. Here, we define $d_{p}$ as the effective length of the electric dipole associated with the generation of electron–hole pairs and excitons, which is also linked to the Bohr exciton radius. Again, we use Wien’s law to determine the temperature θ

(150) $$\theta \;l_{0}=C_{w},$$

where $l_{0}$ is a characteristic width on the nanometer scale, a crucial parameter for elucidating the system’s behavior.

### 9.1. Monotonic Behavior

Monotonic behavior is observed in multi-layer two-dimensional semiconductors. For these systems, the transient dipole length should be related to the average layer width, $d_{p}=\kappa N\bar{l}\left(T\right)$, where κ is a proportionality constant and *N* is the number of layers.

Determining the average layer width, $\bar{l}\left(T\right)$, necessitates a microscopic model. In this framework, $\bar{l}\left(T\right)$ is linked to the deformation relative to the layer width at the minimum experimental temperature, $l_{0}$, denoted as $u=l\left(T\right)-l_{0}$. Here, we assume a model such that this deformation arises from a linear chain of atoms interacting unharmonically through a Morse potential [75]

(151) $$V\left(u\right)=D{\left[1-e^{-au}\right]}^{2}.$$

For this potential, the average system deformation can be expressed in terms of the vibrational temperature of phonons $T_{v}=h\nu_{phon}/k_{B}$, which is associated with a characteristic frequency $\nu_{phon}$:

(152) $$\bar{u}=3ba^{-1}\frac{\bar{E}\left(T\right)}{k_{B}T_{v}}.$$

Here, $b=k_{B}T_{v}/\left(4D\right)$ is a parameter representing the degree of anharmonicity inherent in the Morse interaction. This parameter is expressed as the ratio of the vibrational energy $k_{B}T_{v}$ to the interatomic interaction strength *D*. Additionally, $a^{-1}$ denotes the range of the interatomic potential. A higher vibrational energy corresponds to greater anharmonicity and an increased value of *b*. Thus, the term $3ba^{-1}$ serves as a measure of the range of the potential while accounting for its anharmonicity. Finally, $\bar{E}\left(T\right)$ represents the average internal energy of a harmonic oscillator, that is

(153) $$\bar{E}\left(T\right)=\frac{k_{B}T_{v}}{e^{\frac{T_{v}}{T}}-1}.$$

From the two preceding equations, we can deduce the explicit expression for the averaged layer width as

(154) $$\bar{l}\left(T\right)=l_{0}\left\{+\frac{3ba^{-1}}{l_{0}}\frac{1}{e^{\frac{T_{v}}{T}}-1}\right\}.$$

Therefore, by applying this last equation to the definition of $d_{p}$, and using Equation (149), we find the desired result for the gap energy:

(155) $$E_{g}=E_{g,0}+\frac{hc}{C_{w}}\frac{1}{\kappa N}\frac{T}{1+\frac{\lambda }{e^{\frac{T_{v}}{T}}-1}},$$

where we have defined the length ratio $\lambda =3ba^{-1}/l_{0}$, which compares the effective range of the potential, $3ba^{-1}$, to the characteristic length of the material (such as the width in the case of 2-D materials), $l_{0}$, at the minimum experimental temperature $T_{0}$. In this context, $E_{g,0}$, denotes the reference energy at the temperature $T_{0}$.

For 2-D materials, it is reasonable to assume that $3ba^{-1}≲l_{0}$ which suggests that the term

(156) $$\frac{\lambda }{e^{\frac{T_{v}}{T}}-1}$$

in Equation (155) can have a significant impact.

We emphasize that Equation (155) represents one of our most remarkable results. It captures the dependence of the bandgap energy on both the number of layers and temperature, specifically predicting a monotonic increase as the temperature rises. Systems where our mathematical model holds true allow for this.

**Figure 14:**
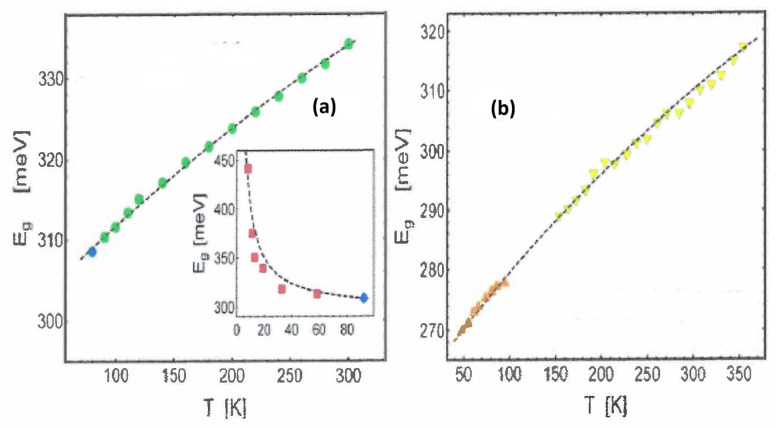
(**a**) Gap energy as a function of the temperature for a sample with 91 layers (∼46 nm). Symbols correspond to experimental data taken from [76] and the dashed black line is the fit using Equation (155). We have assumed that the vibrational temperature is $T_{v}=80$ K, that is the crossover temperature of the gap energy and λ=0.11. The proportionality factor assumed is κ=0.0315 and therefore, κN=2.9. The inset shows the fit (black dashed line) of the experimental data taken from [76] (red squares) of the gap energy as a function of the layer number *N* at 80 K. The fit has the same values of the parameters used in the main figure. (**b**) Gap energy as a function of the temperature, data taken from Ref. [77]. The value of the anharmonicity parameter was λ=0.11 and κN=2.1. Reproduced from Ref. [74] [doi: 10.1039/d4cp01772b], with permission from the Royal Society of Chemistry.

We demonstrate the accuracy of our theory through a comparison with experiments, as depicted in the two plots of Figure 14.

The crossover temperature of the gap energy, as mentioned in the caption of Figure 14, corresponds to an effect observable in Ref. [76] when considering a strictly linear temperature dependence of the gap energy.

### 9.2. Non-Monotonic Behavior

In contrast to the 2-D materials discussed above, experimental observations show that the bandgap energy of 3-D nanocristals displays non-monotonic behavior as the temperature increases, showing both blueshift and redshift. The non-monotonic behavior mentioned is also captured by our Equation (155). Consider the case where λ≲1, suggesting that the range of the interatomic potential may be smaller than the characteristic length. Given this scenario, the Bose-like factor in Equation (156) can be considered a small parameter, enabling an expansion of Equation (155) based on this parameter. By performing the mentioned expansion up to the first order, the energy gap can be represented by the following relation

(157) $$E_{g}=E_{g,0}+\frac{hc}{C_{w}}\frac{1}{\kappa^{*}}T\left(1-\frac{\delta }{e^{\frac{T_{v}^{*}}{T}}-1}\right).$$

In Equation (157), we have redefined the set of parameters as $\kappa^{*}$, δ and $T_{v}^{*}$ to distinguish them from their counterparts κ, λ and $T_{v}$ in Equation (155), as both Equation (155) and Equation (157) describe systems with differing characteristics.

**Figure 15:**
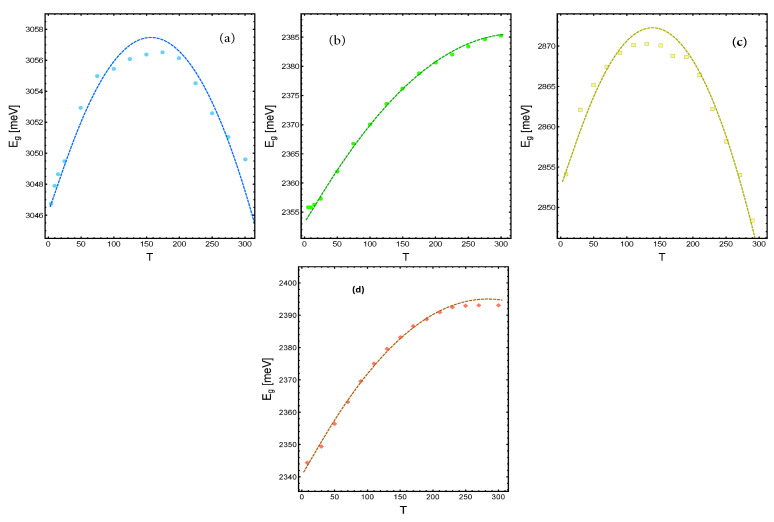
(**a**) PL peak energy position versus temperature of CsPbCl_3_ nano-crystals. The symbols represent experimental data taken from [78]. (**b**) PL peak energy position versus temperature of the experimental for CsPbBr_3_, data taken from [78]. (**c**) PL peak energy position versus temperature of CsPbBr_3_ nanocrystals built by the superposition of several atomic layers (symbols) [79]. (**d**) Data and fit of PL peak energy position versus temperature of two monolayer-thick nanoplatelet films of CsPbBr_3_, data (symbols) taken from [79]. All fits were done using Equation (157). Reproduced from Ref. [74] [doi: 10.1039/d4cp01772b], with permission from the Royal Society of Chemistry.

In Figure 15a, we show the peaks of the PL of CsPbCl_3_ nano-crystals at different temperatures. They display an initial blueshift followed by a redshift. The data were taken from Ref. [78]. The change of trend has been interpreted by the authors as a structural phase change in the crystal at T∼175−200 K. The parameters used in the fit were δ=0.78 (similar to the value of λ for the case of BP) and $\kappa^{*}=3.5$, also consistent with the value used for κN in the BP case. The vibrational temperature used was $T_{v}=175$ K.

In Figure 15b, we show the PL peak energy position versus temperature of CsPbBr_3_ nanocrystals build through a solution-phase synthesis [80]. The data were taken from [78]. The parameters used in the fit were δ=0.34, $\kappa^{*}=2.33$ and $T_{v}=175$ K.

Similarly, in Figure 15c, the data and fit of PL peak energy position versus temperature of two monolayer-thick nanoplatelet films of CsPbBr_3_ are shown. The parameters used in the fit were δ=0.9, $\kappa^{*}=1.89$ and $T_{v}=175$ K.

In Figure 15d, we show the PL peak energy position versus temperature of CsPbBr_3_ nanocrystals build by a superposition of several atomic layers. The data were taken from [79]. The parameters used in the fit were δ=0.39, $\kappa^{*}=0.79$ and $T_{v}=175$ K.

## 10. PL Spectrum [74]

The electronic transitions between conduction and valence bands, considered as a collection of random events between states in two energy bands occurring randomly in time at a constant average rate, give rise to photon emission that follows a shot noise process. This process is mathematically described by the Poisson distribution [81,82]

(158) $$P\left(k,\alpha \right)=e^{-\alpha }\frac{\alpha^{k}}{k!}.$$

Within this framework, $k=E/\epsilon_{0}$, is the random variable representing the number of electronic transitions with energy *E*, (as detailed in Equation (125)), and $\alpha =E_{g}/\epsilon_{0}$ is the ratio between the mean energy $E_{g}$ of these transitions to $\epsilon_{0}$, the characteristic energy separation between adjacent states within a band. This formulation assumes that the transition energy *E* occurs in discrete steps of size $\epsilon_{0}$.

In the limit of large α, the Poisson distribution defined in Equation (158) approximates a Gaussian distribution, a well-established result from the central limit theorem. Hence, one can write

(159) $$PL_{E}≃Q\left(T\right)e^{-\frac{{\left(E-E_{g}\right)}^{2}}{2\epsilon_{0}E_{g}}},$$

where the full width at half maximum (FWHM) is proportional to $E_{g}$. In practice, Q(T) is used as a normalization factor of the PL spectrum. Physically, this Q(T) is likely related to two different processes, the partial absorption of the luminic irradiation by the material wrapping the photoluminescent source and to the electron–phonon interaction.

**Figure 16:**
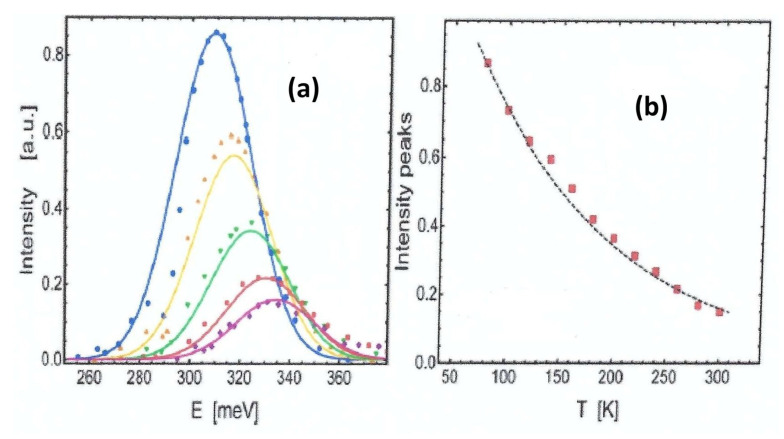
(**a**) Photoluminescence (PL) spectra of black phosphorene as a function of the emission energy for different control temperatures *T*. Symbols are data taken from Ref. [76] and solid lines correspond to the fit using Equations (159)–(161). (**b**) Intensity peaks as a function of the temperature. Symbols are experimental data taken from Ref. [76], whereas the dashed line is a fit using Q(T) given by Equation (161). Reproduced from Ref. [74] [doi: 10.1039/d4cp01772b], with permission from the Royal Society of Chemistry.

An explicit formula for Q(T) is needed to complete our analysis of the PL spectrum. We proceed by assuming a specific behavior for the fractional decrement of the peak intensity:

(160) $$-\frac{dQ(T)}{Q(T)}∼\frac{dT}{T_{v}},$$

where $T_{v}$ is assumed to be the phonon temperature for convenience. Up to the lowest order, the previous relation leads to

(161) $$Q\left(T\right)=Q_{0}exp\left[-\gamma T/T_{v}\right],$$

where $Q_{0}$ and γ are free parameters. Using Equation (161), we obtain Figure 16 which represents the photoluminescence spectra of BP taken from [76] as a function of the emission energy *E* for different control temperatures *T*.

## 11. Conclusions

This review establishes the Finite-size Statistical Mechanics theory as a powerful, unifying framework. We demonstrated its potential across a broad spectrum of intriguing physical systems, successfully applying it to both non-equilibrium steady states and materials under quantum confinement. A cornerstone of this success is the theory’s ability to model the thermal and optoelectronic properties of nanomaterials by revealing the underlying effects of quantum confinement. The theory’s core strength lies in exploiting the invariance of the statistical mechanics description under energy and temperature rescaling—a versatile procedure that extends its utility far beyond equilibrium, as evidenced by its application to non-thermodynamic equilibrium systems ([18,19,20,24]). In the vast majority of cases presented, our theoretical predictions have been rigorously validated by experimental data reported in the referred literature.

## Figures and Tables

**Figure 9:**
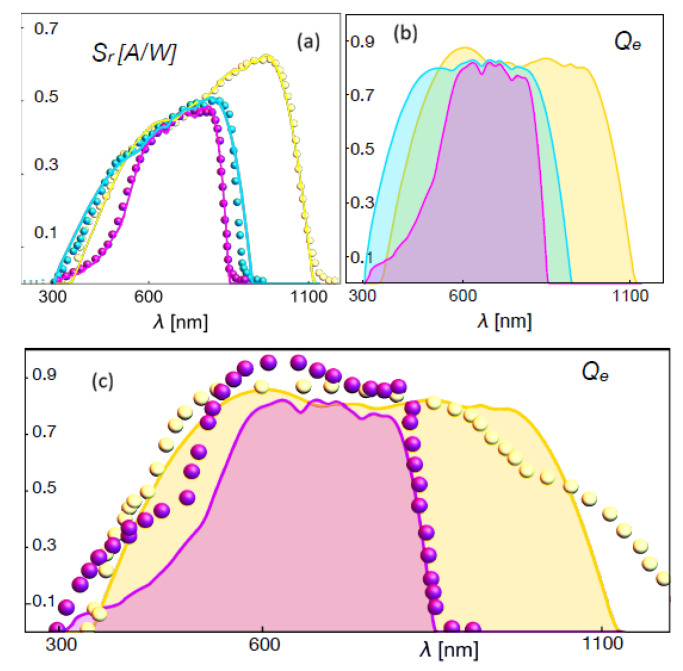
(**a**) Spectral response Sr, Equation (116), and (**b**) quantum efficiency, Equation (118), of different PVCs [53] as a function of the wavelength for 1.5AMG. The yellow symbols and lines correspond to a CIGS cell. The cyan symbols and lines correspond to GaAs-based and filtered cells and the magenta symbols and lines to a CdTe-based cell. (**c**) Comparison of the quantum efficiencies, inferred by using Equation (118), from the fit of CIGS and CdTe data from [53] with independent data from [54]. Reproduced from Ref. [43].

## Data Availability

No new data were created or analyzed in this study.
